# The HIV-1 capsid core is an opportunistic nuclear import receptor

**DOI:** 10.1038/s41467-023-39146-5

**Published:** 2023-06-24

**Authors:** Guangai Xue, Hyun Jae Yu, Cindy Buffone, Szu-Wei Huang, KyeongEun Lee, Shih Lin Goh, Anna T. Gres, Mehmet Hakan Guney, Stefan G. Sarafianos, Jeremy Luban, Felipe Diaz-Griffero, Vineet N. KewalRamani

**Affiliations:** 1 Model Development Section, Cancer Innovation Laboratory, National Cancer Institute, Frederick, MD 21702 USA; 2 Basic Science Program, Leidos Biomedical Research, Frederick National Laboratory, Frederick, MD 21702 USA; 3 Department of Microbiology and Immunology, Albert Einstein College of Medicine, Bronx, NY 10461 USA; 4 Program in Molecular Medicine, University of Massachusetts Medical School, Worcester, MA 01605 USA; 5 Bond Life Sciences Center, Chemistry, University of Missouri, Columbia, MO 65201 USA; 6 Bond Life Sciences Center, MMI, Biochemistry, University of Missouri, Columbia, MO 65201 USA; 7 Laboratory of Biochemical Pharmacology, Department of Pediatrics, Emory University School of Medicine, Atlanta, GA 30322 USA

**Keywords:** HIV infections, Retrovirus

## Abstract

The movement of viruses and other large macromolecular cargo through nuclear pore complexes (NPCs) is poorly understood. The human immunodeficiency virus type 1 (HIV-1) provides an attractive model to interrogate this process. HIV-1 capsid (CA), the chief structural component of the viral core, is a critical determinant in nuclear transport of the virus. HIV-1 interactions with NPCs are dependent on CA, which makes direct contact with nucleoporins (Nups). Here we identify Nup35, Nup153, and POM121 to coordinately support HIV-1 nuclear entry. For Nup35 and POM121, this dependence was dependent cyclophilin A (CypA) interaction with CA. Mutation of CA or removal of soluble host factors changed the interaction with the NPC. Nup35 and POM121 make direct interactions with HIV-1 CA via regions containing phenylalanine glycine motifs (FG-motifs). Collectively, these findings provide additional evidence that the HIV-1 CA core functions as a macromolecular nuclear transport receptor (NTR) that exploits soluble host factors to modulate NPC requirements during nuclear invasion.

## Abstract (web summary)

Nuclear import of the HIV-1 capsid (CA) is mediated through direct interactions with components of the nuclear pore complexes. Here, the authors identify Nup35 and POM121 as HIV-1 CA interacting factors regulating nuclear entry and further demonstrate regulation of the process by soluble factors Cyclophilin A and CPSF6.

## Introduction

HIV-1 exhibits significant flexibility in its utilization of the NPC to access the nucleus during early replication steps. Comprised of over 30 distinct proteins, the NPC incorporates six subcomplexes based on stoichiometry and distribution within the NPC (Supplementary Fig. 1a)^1^. Approximately one-third of Nups contain FG-dipeptides, motifs which are targets for NTR interaction for directional movement of cargo from the cytoplasm to the nucleus^2^. FG-dipeptides in a subset of Nups are embedded in prion-like low complexity regions (LCRs) that facilitate multivalent engagement by the HIV-1 CA lattice^3^. Genetic and biochemical studies have implicated the FG-Nups Nup153 and Nup358 as contributing to HIV-1 nuclear entry^4–10^. These Nups decorate opposite poles of the NPC with tentacles of Nup358 extending into the cytoplasm from the outer nuclear membrane and Nup153 projecting a basket-like structure from the inner nuclear membrane into the nucleoplasm. Forced dimerization of Nup62 induces a formidable block to HIV-1 nuclear entry highlighting the centrality of the NPC channel to virus infection^11^. HIV-1 with mutations in CA that prevent interaction with soluble factors such as CPSF6 and CypA, exhibit a differential dependence on Nups during infection, including loss of reliance on both Nup153 and Nup358. CypA interacts with CA via the P90 residue exposed on loop in the N-terminal domain (NTD), and CPSF6 interacts via the N74 pocket within an NTD and C-terminal domain (CTD) interface present in hexameric CA in the virion core (Supplementary Fig. 1b, c). P90A and N74D mutations, respectively, impair CypA and CPSF6 interactions with CA. We considered the possibility that CPSF6 and CypA binding to HIV-1 in the cell cytoplasm affected subsequent interactions at the NPC and thus routes of nuclear entry.

## Results

**Fig. 1:**
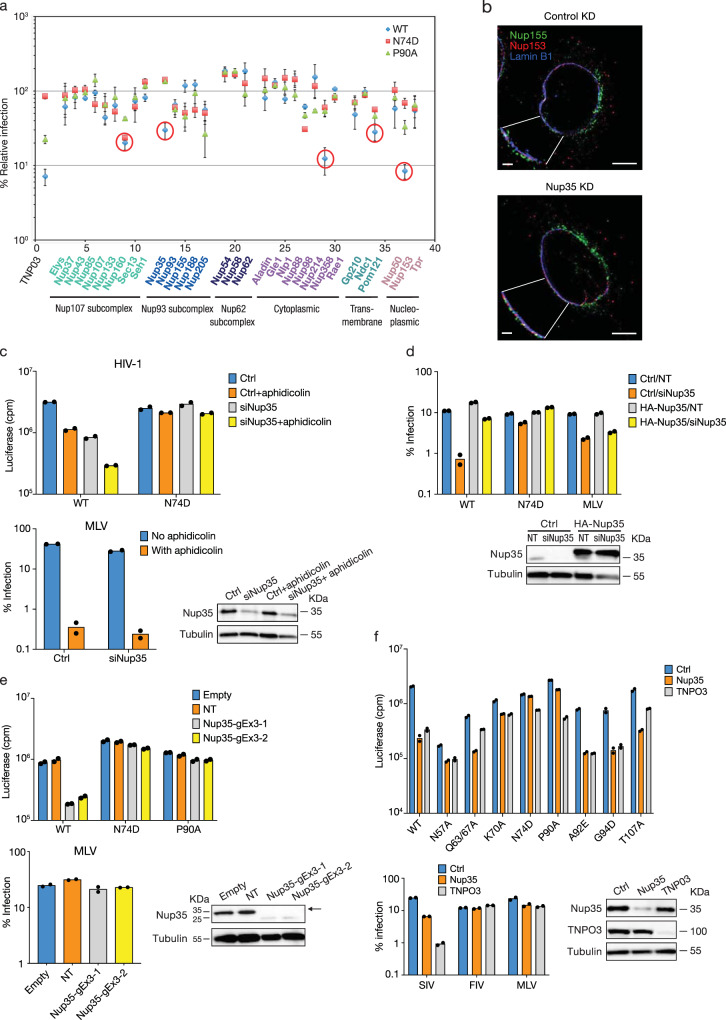
HIV-1 dependence on Nup35. **a** CA-dependent use of nucleoporins by HIV-1. Wild-type (WT) or CA-mutant HIV-1-RFP reporter virus infection of control or target siRNA transfected HeLa cells. Nups targeted by siRNAs are grouped by NPC subcomplexes. WT HIV-1 infection decreases of greater than 3-fold after knockdown of nucleoporins are encircled. Percent relative infection is mean ± s.d., *n* = 2–5 for nucleoporins, *n* = 15 for TNPO3 from biologically independent experiments. **b** Distribution of Nup153 and Nup155 after Nup35 knockdown. Localization of Nup153 (red), Nup155 (green), and Lamin B1 (blue) in HeLa cells by 3D SIM after immunostaining. Representatives of 40 cells from two independent experiments. A z-section image is presented. Scale bars: 5 µm (overview), 1 µm (inset). **c** WT HIV-1 or N74D HIV-1 or MLV infection of dividing and non-dividing (2 μg/ml aphidicolin-treated) control or Nup35 siRNA transfected HeLa cells. *n* = 2 technical replicates, representative of two independent experiments. Western blotting confirmed knockdown efficiency. **d** HeLa cells stably expressing human HA-Nup35 lacking 3′-UTR or transduced with control vector (LPCX-HA), were transfected with non-target (NT) siRNA or Nup35 siRNA targeting 3′-UTR region for 48 h, then infected with VSV-G-pseudotyped viruses. *n* = 2 technical replicates, representative of three independent experiments. Western blot analysis confirmed Nup35 restoration. **e** HIV-1 CA determines Nup35 dependence in HeLa knockout cell clones. WT HIV-1 or CA-mutant HIV-1 or MLV infection of Nup35 knockout HeLa cell clones. *n* = 2 technical replicates, representative of three independent experiments. Western blots show the knockout efficiency of Nup35. Empty, cell lines transiently co-transfected with pX330 and a puromycin-expression vector; NT, non-targeting control lines lacking a gene-specific gRNA. **f** WT HIV-1 or CA-mutant HIV-1 or various retroviruses infection of control or target siRNA transfected HeLa cells. *n* = 2 technical replicates, representative of two independent experiments. Western blotting confirmed knockdown efficiency. Source data are provided as a Source Data file.

To this end, we performed a genetic screen with small-interfering RNAs (siRNAs) targeting 32 human Nups in HeLa cells to identify those that exhibited CA-dependence during infection with VSV-G pseudotyped HIV-1 vectors encoding red fluorescent protein (RFP). As a positive control to inhibit infection of HIV-1 with wild-type (WT) CA, TNPO3 was also depleted using siRNA^4^. Parallel infections were performed with HIV-1 CA mutant (N74D and P90A) virus vectors and Moloney murine leukemia virus (MLV) vectors. Nup35, Nup153, Nup358, and POM121 depletion were observed to inhibit WT HIV-1 infection but affected N74D and P90A HIV-1 infection to a lesser degree (Fig. 1a). Nup35 and POM121 have not previously been studied as potential HIV-1 cofactors, and notably, their depletion had little effect on MLV infection (Supplementary Fig. 2). As has been previously reported^8,12^, Nup160 depletion affected WT and CA mutant HIV-1 infection, which is likely due to the critical role it plays in NPC formation.

By contrast, depletion of Nup62 or Nup214 elevated WT HIV-1 infection, and Nup54 and Nup58 knockdown enhanced infection of both WT and CA mutant HIV-1. MLV infection was not increased in Nup54, Nup58, Nup62, or Nup214 knockdown cells (Supplementary Fig. 2). Nup54, Nup58, and Nup62 are notable due to their formation of an FG-network that provides a selective barrier to transport between cytoplasm and nucleus.

Because Nup35 and POM121 depletion affected WT HIV-1 infection but not CA mutant HIV-1 in a pattern similar to Nup153 and Nup358, we focused efforts on these two Nups. Initial studies were performed with Nup35 as it was less essential to N74D and P90A HIV-1 relative to POM121. Nup153 was selected over Nup358 as a control in subsequent experiments given significant cellular toxicity observed after Nup358 knockdowns. Nup153 was also of interest because HIV-1 CA engages it via an FG-motif present within a prion-like LCR^6,13^.

To determine whether the effect of Nup35 on WT HIV-1 infection was direct, we sought to examine the stability of other Nups after Nup35 depletion with siRNA. Knockdown of Nup35 did not affect levels of other Nups present in the Nup93 subcomplex of which it is a member nor did it affect levels of TNPO3, Nup153, or Nup358 (Supplementary Fig. 3). These data suggested that the indirect degradation of other Nups, including those known to interact with HIV-1, did not account for the infection block. Previous studies have shown that Nup35 contributes to NPC assembly in xenopus eggs^14^. In order to understand whether the inhibition of HIV-1 infection was due to impaired NPC assembly in human cells, we performed structured illumination microscopy (SIM). Nup153 remained associated with the nuclear membrane in Nup35 knockdown-HeLa cells as did the Nup93 subcomplex constituent Nup155 (Fig. 1b). These observations were consistent with a previous study showing that depletion of Nup35 did not alter Nup153 localization^15^. We then measured HIV-1 infectivity in cells arrested at the G1/S transition of the cell-cycle. WT HIV-1 infectivity was further inhibited in growth-arrested Nup35 knockdown cells. By contrast, N74D HIV-1, which requires NPCs for infection, was not affected by the cell-cycle arrest in the knockdown cells. MLV, which requires nuclear membrane breakdown to access host chromatin, was almost completely abolished for infection (Fig. 1c). Taken together, these results suggest that transient Nup35 loss was specific in effect and not due to a loss of NPCs.

We next tested three unique Nup35 siRNAs from the mixture used in the screen separately. Consistent with the pooled siRNA results, two different siRNAs directed against Nup35, siRNA-2 and siRNA-3, reduced WT HIV-1 infection approximately 10-fold (Supplementary Fig. 4a) correlating with Nup35 depletion, whereas N74D HIV-1 and P90A HIV-1 were resistant to Nup35 depletion.

To test whether the reductions in HIV-1 infection were due to off-target effects, we performed a functional rescue experiment (Fig. 1d). Nup35 siRNA-3 targets 3′-UTR sequence. Thus HeLa cells were transduced either with LPCX empty vector or with LPCX vector expressing HA-tagged Nup35 that lacks the 3′-UTR and is therefore resistant to silencing by the siRNA. Western blot analysis confirmed that siRNA knockdown reduced the endogenous level of Nup35 in both transduced cell populations; however, ectopically expressed HA-Nup35 was unaffected by Nup35 siRNA treatment. Consistent with prior results, cells depleted for endogenous Nup35 and transduced with the LPCX empty vector were more than 10-fold less permissive to HIV-1 infection. In contrast, Nup35 siRNA-treated cells that expressed HA-Nup35 remained susceptible to WT HIV-1. N74D HIV-1 infection was relatively consistent under the different Nup35 expression conditions. Notably, the small infection decrease in MLV infection occurring after Nup35 knockdown was not offset in cells expressing exogenous HA-Nup35 suggesting the effect on MLV could be indirect.

CRISPR/Cas9 gene-editing of *nup35* in HeLa cells confirmed the siRNA findings. While 5 different guide RNAs (gRNAs) were tested, only one targeting the third exon of *nup35* yielded cell clones deficient of full-length Nup35. These clones uniformly expressed a truncated form of Nup35 that likely initiated from an in-frame methionine at coding residue position 62 of Nup35 within exon 3 based on the protein size and sequencing analysis (Fig. 1e and Supplementary Fig. 4b). The internally initiated form of Nup35 may have permitted cell survival. These cells expressing the suspected N-terminally truncated Nup35 had reduced susceptibility to HIV-1 infection but not N74D or P90A HIV-1 infection (Fig. 1e). Consistent with siRNA knockdown results, knockout of full-length Nup35 did not affect levels of other Nups present in the Nup93 subcomplex nor did it affect the levels of TNPO3, Nup153, or Nup358 (Supplementary Fig. 4c). Furthermore, the functional rescue experiments showed that ectopically expressed Nup35 rescued WT HIV-1 infection, but not N74D HIV-1 infection in Nup35-knockout cell clones. By contrast, MLV infection was not affected by ectopically expressed Nup35 (Supplementary Fig. 4d).

Because N74D HIV-1 and P90A HIV-1 were insensitive to Nup35 depletion, we examined other viruses with CA mutations that influence interaction with CPSF6 or CypA for infection of Nup35 knockdown cells. HIV-1 with N57A, Q63A/Q67A, K70A, or T107A mutations in CA are reduced in sensitivity to CPSF6-mediated infection blocks and are similarly diminished in binding CPSF6^16^. Relative to WT HIV-1, these viruses are also less sensitive to TNPO3 depletion which enables CPSF6 inhibition of infection (Fig. 1f). While N57A and K70A HIV-1 were less sensitive to Nup35 depletion, Q63A/Q67A and T107A HIV-1 resembled WT HIV-1 in infection indicating that CPSF6-interaction on its own does not predict Nup35 dependence.

In contrast to WT HIV-1, A92E HIV-1 and G94D HIV-1 replicate more efficiently in the absence of CypA^17,18^. These mutant viruses retain CypA and CPSF6 interaction sites. Similar to WT HIV-1, these viruses were impaired for infection of Nup35 and TNPO3 knockdown cells (Fig. 1f). We also tested simian immunodeficiency virus from macaques (SIV_mac239_), which replicates independent of CypA interaction, and feline immunodeficiency virus (FIV) which does not interact with either CypA or CPSF6. SIV_mac239_, which retains the conserved region necessary for CPSF6 interaction, was potently impaired by TNPO3 knockdown but exhibited reduced dependence on Nup35 (Fig. 1f). FIV was unaffected by either Nup35 or TNPO3 knockdown. Taken together, these results demonstrate that HIV-1 CA determines sensitivity to Nup35 depletion, and CypA interaction can modulate the Nup35-dependence.

### CypA impairs HIV-1 infection in Nup35-knockdown cells

**Fig. 2:**
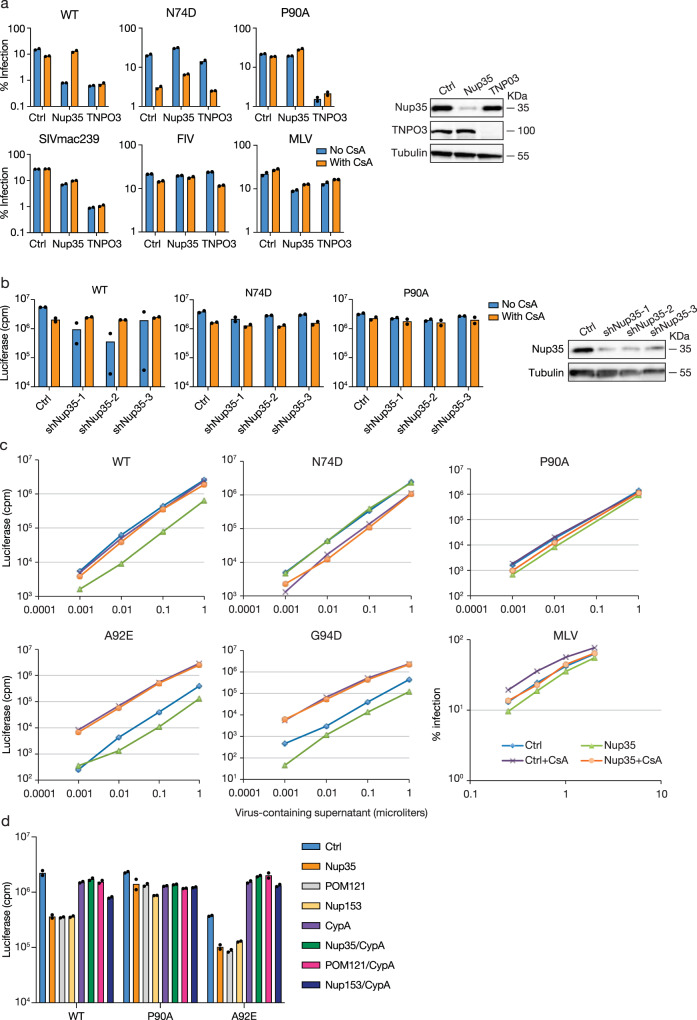
HIV-1 dependence on Nup35, Nup153, and POM121 is regulated by CypA. **a** HIV-1, SIV, FIV, and MLV infection in the absence or presence of CsA in Nup35-depleted cells. WT HIV-1 or CA-mutant HIV-1 or various retroviruses infection of control or target siRNA transfected HeLa cells in the presence or absence of cyclosporin (CsA). *n* = 2 technical replicates, representative of two independent experiments. Western blotting confirmed knockdown efficiency. **b** CsA restores HIV-1 infection in Nup35-depleted MT4 cells. WT HIV-1 or CA-mutant HIV-1 infection of control or target shRNA transduced MT4 cells in the presence or absence of CsA. *n* = 2 technical replicates, representative of two independent experiments. Western blot analysis confirmed Nup35 depletion by three different shRNAs. **c** CsA restores A92E and G94D HIV-1 infectivity in Nup35 knockdown cells. WT HIV-1 or CA-mutant HIV-1 or HIV-1 or MLV infection of control or Nup35 siRNA transfected HeLa cells in the presence or absence of CsA. *n* = 2 technical replicates, representative of three independent experiments. **d** CypA depletion by siRNA restores HIV-1 infectivity in Nup35 and POM121 knockdown HeLa cells. WT HIV-1 or CA-mutant HIV-1 infection of control or single-knocked down or double-knocked down HeLa cells. *n* = 2 technical replicates, representative of three independent experiments. Source data are provided as a Source Data file.

To directly test whether CypA was required for the HIV-1 dependence on Nup35, we treated Nup35-knockdown cells with cyclosporine A (CsA) to inhibit the CypA-CA interaction (Fig. 2a). WT HIV-1 infectivity was rescued to the level of control cells by CsA treatment in Nup35-knockdown cells. In contrast, CsA had minor effects on WT HIV-1 infection in either control cells or TNPO3-knockdown cells. N74D HIV-1 infection was previously demonstrated to be sensitive to CsA treatment^19^, and it remained sensitive in Nup35- and TNPO3-knockdown cells. As expected, the infectivity of HIV-1 with the CypA binding mutation P90A was not affected by CsA treatment in any cell type. These data demonstrate that CsA restores HIV-1 replication following Nup35 knockdown. The effect was specific to HIV-1, as SIV_mac239_, FIV, and MLV were not affected by CsA in Nup35-depleted cells (Fig. 2a). Taken together, these data indicate that the block to HIV-1 infection in Nup35-depleted cells is dependent on the virus binding CypA.

We sought to understand whether CypA affected WT HIV-1 dependence on Nup35 in the T cell line, MT4, which are highly permissive to transduction with shRNA-expressing lentiviral vectors. Cells were transduced with GFP-encoding shRNA vectors targeting Nup35 and then challenged with HIV-1 in the absence and presence of CsA (Fig. 2b). While knockdown of Nup35 specifically diminished WT HIV-1 infection in 2 of 3 shRNA lines, CsA treatment restored WT HIV-1 infectivity to levels observed in shRNA control cells treated with the drug. These data underline the pivotal role of CypA in determining the HIV-1 sensitivity to Nup35 depletion.

Given the interrelation between CypA binding and HIV-1 sensitivity to Nup35 depletion, we considered whether CsA-dependent viruses, A92E and G94D HIV-1, would remain sensitive to Nup35 depletion after CsA treatment. The A92E and G94D HIV-1 infection block in HeLa cells mediated by CypA occurs at nuclear entry^20^. When faced with a different block to nuclear entry in TNPO3-depleted cells, CsA treatment fails to fully restore the replication of these viruses (Supplementary Fig. 5a). A92E and G94D HIV-1 were also additionally sensitive to loss of Nup35 in cells (Fig. 2c). However in the presence of CsA, A92E and G94D HIV-1 infectivity in Nup35 knockdown cells rises to the level of the mutant viruses in control cells treated with CsA. Collectively, these data suggest the mechanisms underlying HIV-1 infection inhibition in Nup35-knockdown cells may be similar to the infection blocks encountered by A92E and G94D HIV-1 in normal cells in that CypA drives these viruses toward a nuclear entry pathway that they are unable to utilize.

### HIV-1 infection in POM121 and Nup153 knockdown cells is affected by CypA

The degree by which CypA controlled HIV-1 sensitivity to Nup35 depletion led us to investigate whether HIV-1 interactions with POM121 and Nup153 were CypA-dependent. Cells were either individually treated with siRNAs targeting Nup35, POM121, Nup153, and CypA, or doubly treated with siRNAs targeting Nup35, POM121, and Nup153 in combination with siRNA targeting CypA. Notably, depletion of CypA not only restored HIV-1 infection in Nup35-knockdown cells but similarly restored infection in POM121-knockdown cells and partly restored infection in Nup153-knockdown cells (Fig. 2d). A92E HIV-1 was also largely insensitive to the different Nup knockdowns in cells in which CypA was also depleted. By contrast, MLV infection was mostly unaffected in cells with single Nup knockdowns or Nup knockdowns in combination with CypA knockdowns (Supplementary Fig. 5b).

### Nup35 knockdown impairs HIV-1 nuclear entry

**Fig. 3:**
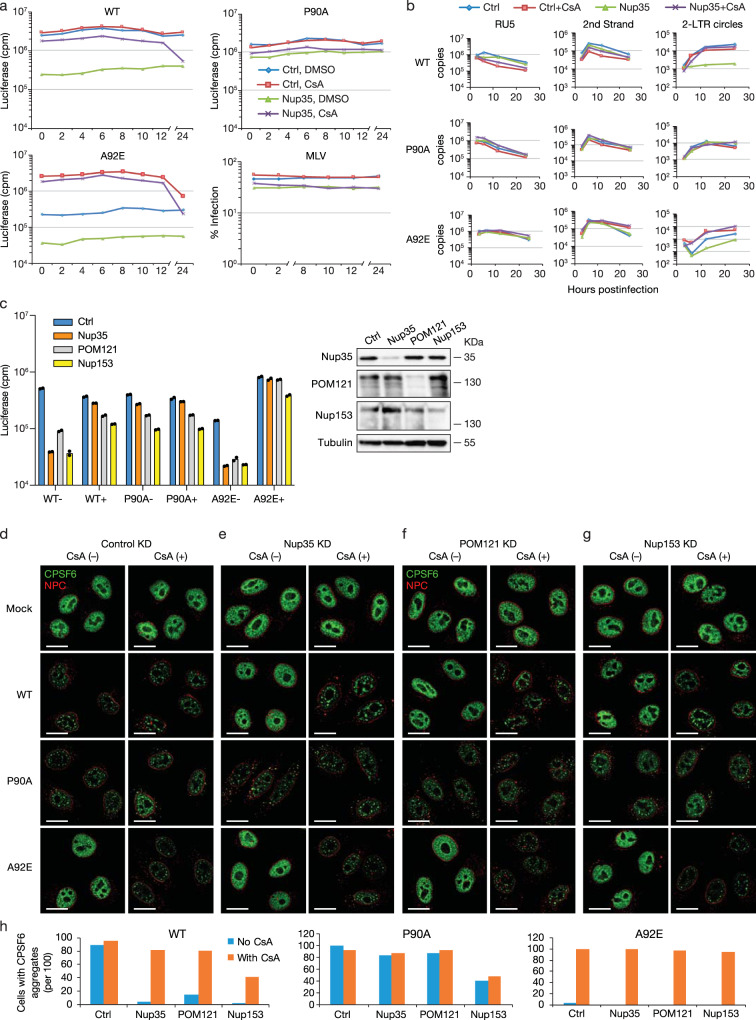
Nup35 knockdown impairs HIV-1 nuclear entry. **a** CsA can restore HIV-1 infectivity in Nup35 knockdown cells 12 h after virus challenge. WT HIV-1 or CA-mutant HIV-1 or MLV infection of control or Nup35 siRNA transfected HeLa cells in the presence or absence of CsA. *n* = 2 technical replicates, representative of two independent experiments. **b** HIV-1 reverse transcription is not blocked in Nup35 knockdown cells but 2-LTR circle forms are not observed. DNase-treated WT HIV-1 or CA-mutant HIV-1 infection of control or Nup35 siRNA transfected HeLa cells in the presence or absence of CsA. Cell DNA was extracted at 3, 6, 12, and 24 h after infection and used to detect early (RU5) RT, late (2nd strand) RT, and 2-LTR circles (right). *n* = 2-3 technical replicates, representative of two independent experiments. The amount of DNA in each PCR assay was normalized by actin. HIV-1 infection was also measured at 48 h in parallel samples to monitor HIV-1 infectivity (Supplementary Fig. 5c). **c** CsA restores HIV-1 infection in Nup35, Nup153, and POM121 knockdown HeLa cells. WT HIV-1 or CA-mutant HIV-1 infection of control or target siRNA transfected HeLa cells in the presence or absence of CsA. *n* = 2 technical replicates, representative of three independent experiments. Western blotting confirmed knockdown efficiency. −, without CsA; +, with CsA. **d**–**g** CPSF6 distribution of Nup35, POM121, and Nup153 knockdown cells in the presence or absence of CsA. WT HIV-1 or CA-mutant HIV-1 infection of control or target siRNA transfected HeLa cells. After 12 h, cells were fixed and stained with antibodies against CPSF6 and NPC. Scale bar, 15 μm. Representatives of 100 cells from an experiment. **h** The number of cells with or without CPSF6 aggregates were counted from (**d**–**g**). Source data are provided as a Source Data file.

To gain insight into the specific stage of HIV-1 infection at which Nup35 and CsA exert their effects, we performed time course experiments on Nup35-knockdown HeLa cells that were treated with CsA at various times after exposure to virus. Up to 12 h post-infection of Nup35-depleted cells, WT HIV-1 infectivity could be rescued by CsA treatment; however, CsA completely lost rescue ability at 24 h post-infection (Fig. 3a). Similarly, CsA treatment also rescued the infectivity of CsA-dependent HIV-1 mutant A92E 12 h post-infection, but could not rescue infectivity at 24 h post-infection. P90A HIV-1 and MLV infection were not affected by CsA treatment at any time point, which is consistent with the inability of P90A HIV-1 and MLV to bind CypA.

Given the early reversibility of the infection block and the alteration of the nuclear pore complex by the various Nup knockdowns, we evaluated HIV-1 reverse transcription in cells depleted of Nup35 in the absence or presence of CsA (Supplementary Fig. 5c and Fig. 3b). We observed no difference in the accumulation of early or late reverse transcription products in control cells vs. Nup35-knockdown cells (Fig. 3b). Notably, both WT and A92E HIV-1 exhibited significantly reduced levels of 2-LTR circle junction forms of viral DNA (vDNA) unless treated with CsA. Although 2-LTR circular vDNA represents an abortive, noninfectious path, they form in the nucleus in the presence of DNA Ligase IV^21^. These data indicate that the Nup35 knockdown prevents HIV-1 nuclear entry in the presence of CypA.

We next tested whether CypA also regulated HIV-1 nuclear entry in cells depleted of POM121 or Nup153 using an assay based on nuclear CPSF6 distribution in HIV-1 infected cells^22,23^. As shown by different groups^22,24,25^, HIV-1 complexes with CPSF6 in NPCs and nuclear speckle domains after infection (Supplementary Fig. 6a–c). The aggregation of CPSF6 in nuclear speckles requires interaction with HIV-1 CA^22,24,26^. To microscopically examine the requirements for HIV-1 entering the nucleus, cells were depleted of Nup35, POM121, or Nup153 in the absence or presence of CsA. As before, WT and A92E HIV-1 were sensitive to depletion of these Nups, and this infection reduction could be eliminated through CsA treatment (Fig. 3c). By contrast, MLV infection was not affected by CsA in Nup35, POM121, or Nup153 depleted cells (Supplementary Fig. 6d). CA lattice interactions with CPSF6 induces precipitates, especially in the nucleus where CPSF6 is abundant (Fig. 3d). Although it was in lesser abundance, CPSF6 was observed in the cytoplasm, and more clearly seen in P90A mutant-infected cells (Supplementary Fig. 6e, f). In Nup35-knockdown cells, WT HIV-1 infection no longer leads to CPSF6 precipitation in the nucleus (Fig. 3e). Conversely, CsA treatment recovered CPSF6 aggregation following WT HIV-1 infection. These results further indicate that restoration of HIV-1 infection by CsA is linked to nuclear entry. Similar results were obtained with A92E HIV-1. Notably, this virus did not induce nuclear aggregation of CPSF6 in control cells unless in the presence of CsA. As expected, CsA treatment did not alter CPSF6 distribution in P90A HIV-1 infected control cells, such that CPSF6 aggregates were observed in Nup35-knockdown cells in the absence of CsA. The phenotypes of WT HIV-1, P90A HIV-1, and A92E HIV-1 were consistent in cells depleted of either POM121 (Fig. 3f) or Nup153 (Fig. 3g). Overall levels of CPSF6 remained consistent before and after HIV-1 infection (Supplementary Fig. 6g). These results summarized in Fig. 3h suggest a pivotal role of CypA in regulating HIV-1 nuclear entry in Nup35, Nup153, and POM121 knockdown cells.

### The choreography of HIV-1 interaction with host factors governing nuclear entry reveals a regulatory switch

**Fig. 4:**
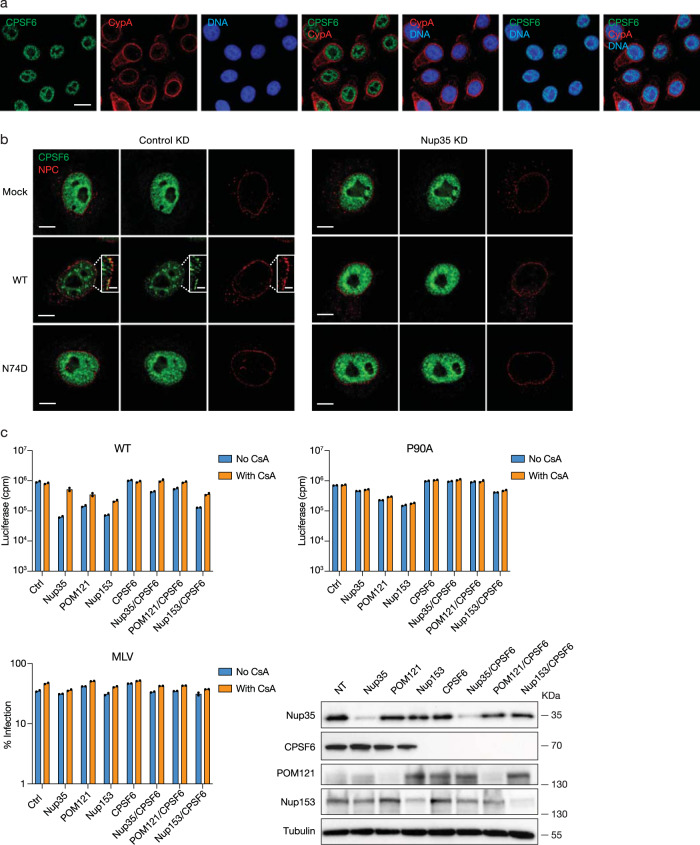
Soluble factors regulate nucleoporin dependence. **a** CypA enrichment at the nuclear periphery. Localization of CPSF6 (green) and CypA (red) in HeLa cells in a z-section image from deconvolution microscopy of immunostained HeLa cells. Scale bar, 15 μm. **b** CPSF6 aggregation at NPC. Control or Nup35 knockdown cells were infected with WT or N74D HIV-1 for 12 h. Localization of CPSF6 (green) and NPC (red) in HeLa cells by 3D SIM after immunostaining. A z-section image is presented. Scale bars: 5 µm (overview), 1.2 µm (inset). **a**, **b** Representative of 50 cells from two independent experiments. **c** Partial restoration of HIV-1 infection in Nup35 and POM121 knockdown cells after CPSF6 depletion. WT HIV-1 or CA-mutant HIV-1 or MLV infection of control or single-knocked down or double-knocked down HeLa cells. *n* = 2 technical replicates, representative of three independent experiments. Western blotting confirmed knockdown efficiency. Source data are provided as a Source Data file.

Given the significant role that soluble factor binding appeared to exert on transport of HIV-1 through the NPC, we considered whether differences in the subcellular localization of CPSF6 and CypA could predict their impact on this process. Although present throughout the cell, CypA is enriched in cytoplasmic compartments, especially in proximity to the nuclear membrane in both HeLa cells and T cell lines (Fig. 4a and Supplementary Fig. 7a-c). This distribution appears relatively stable and is not grossly affected by Nup35-depletion (Supplementary Fig. 7d). CPSF6, consistent with its role in pre-mRNA processing, is predominantly nuclear and distributed in a pattern distinct from CypA and is also unaffected by Nup35 depletion in uninfected cells (Fig. 4b and Supplementary Fig. 7e).

Indeed, in cells depleted of Nup35, POM121, or Nup153, a partial restoration of WT HIV-1 infection is observed after coordinate knockdown of CPSF6 (Fig. 4c), especially in Nup35 and POM121 knockdown cells. HIV-1 infection in these cells can be fully restored after CsA treatment. In comparison, P90A HIV-1 does not interact with CypA, and the infectivity of this virus is slightly diminished in POM121 and Nup153 knockdown cells (Fig. 1a, Fig. 3c, and Fig. 4c). Whereas CsA treatment, predictably, does little to affect P90A infection in the POM121 or Nup153 knockdown cells, CPSF6 depletion potently enhances infection when in conjunction with either knockdown (Fig. 4c). Notably, coordinate depletion of Nup35 and Nup153 potently impaired WT HIV-1 infection (Supplementary Fig. 8a).

We next asked whether CypA was also a key regulator of Nup155 use. Nup155 is a non-FG Nup in the Nup93-subcomplex that we previously noted N74D HIV-1 to be more dependent for infection than WT HIV-1^12^. WT HIV-1 infection is diminished in cells depleted of Nup153 but retains greater infectivity in Nup155-knockdown cells (Supplementary Fig. 8b). Two distinct effects on infection are observed when CypA is depleted in Nup153- and Nup155-knockdown cells. WT HIV-1 infection is increased in the Nup153-knockdown cells as previously noted^27^, and it is decreased in the Nup155-knockdown cells (Supplementary Fig. 8b). The infectivity of WT HIV-1 in the CypA plus Nup155 double knockdown cells is comparable to N74D and P90A HIV-1 infection of the Nup155-depleted cells. In the absence of CypA, it becomes more dependent on Nup155. These data suggest the use of a secondary nuclear pathway for HIV-1 when accessing the Nup153 pathway is not possible.

In order to better understand the interrelation between Nup35- and Nup155-dependent infection, we depleted both factors and assessed the effect of CsA treatment on HIV-1 infection (Supplementary Fig. 8c). As the CypA knockdown data indicated, CsA treatment restores HIV-1 infection in Nup35 knockdown but not Nup155 knockdown cells. In the combined knockdown cells, the loss of CypA interaction only partially restores HIV-1 infection levels, up to the level of what is observed in the Nup155-single knockdown cells. These data further support an important role for Nup155 in HIV-1 nuclear entry distinct from the CypA-dependent path nucleoporins.

### Identification of Nup35 and POM121 domains interacting with HIV-1

**Fig. 5:**
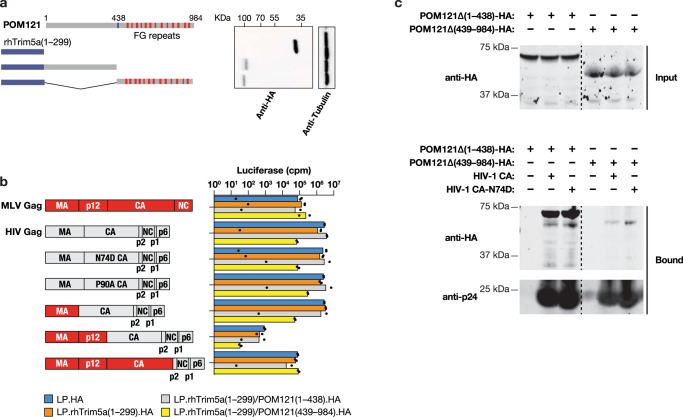
POM121 contributes HIV-1 nuclear import via direct binding to HVI-1 capsid. **a** TRIM5-POM121 inhibition of HIV-1 infection is CA-dependent. **a** Left panel, schematic representation of the rhesus TRIM5 RBCC (RING, B-box2, and coiled coil) domains (1-299) with the POM121 N-terminal fragments (1-438) or C-terminal fragments (439-984). Right panel, western blotting analysis of lysates from HeLa cells expressing TRIM fusion proteins. **b** Left panel, schematic representation of the chimeric HIV/MLV virus. Right panel, MLV or chimeric HIV/MLV virus infection of vector or rhTrim5a.POM121-expressing HeLa cells. *n* = 2 technical replicates, representative of three independent experiments. Western blot analysis of HA-tagged rhTrim5a/POM121 fusion and control constructs in stable HeLa cells. **c** Binding of POM121Δ(1-438) and POM121Δ(438-984) to stabilized in vitro*-*assembled HIV-1 CA complexes. Lysates from transfected HEK293T cells expressing HA-tagged POM121Δ(1-438) or POM121Δ(438-984) (Input) was incubated with HIV-1 CA complexes for 1 h. Capsid complexes were washed two times using capsid binding buffer. Samples were analyzed by western blotting using anti-HA and anti-p24 antibodies (Bound). Similarly, the ability of POM121 variants to bind in vitro in vitro*-*assembled HIV-1 CA complexes bearing the mutation N74D was tested. Similar results were obtained in three independent experiments. Source data are provided as a Source Data file.

We next sought to examine Nup35 and POM121 interaction with HIV-1. We first attempted an approach that has been leveraged in mapping CA-interaction domains in CPSF6 and Nup153, specifically through fusion to the N-terminal RING, B-box 2, and coiled-coil (RBCC) domains of rhesus TRIM5 alpha, substituting the B30.2 (SPRY) domain with a region of interest^6,28^. Attempts to make TRIM5a.Nup35 fusions that were detectable in cell lines were unsuccessful. By contrast, rhTRIM5 fusions to the N-terminus and C-terminus of POM121 were stable (Fig. 5a). We first examined whether HIV-1 and MLV were differentially susceptible to infection in cells expressing the TRIM5a.POM121 fusion proteins (Fig. 5b). Whereas MLV displayed no apparent sensitivity to the different TRIM5a.POM121 proteins, HIV-1 (LAI Gag) was specifically inhibited by a TRIM5 fusion to the FG-rich, C-terminal domain of POM121. Moreover, testing with HIV-1/MLV Gag chimeric viruses revealed that HIV-1 CA was required for sensitivity to this protein. We extended this analysis to the CA-mutant HIV-1 (NL4-3 Gag) isolates (Supplementary Fig. 9a). Although HIV-1 CA governs susceptibility to TRIM5a.POM121 C-terminal fusion, the N74D and P90A mutations were insufficient to escape restriction.

To investigate whether POM121 associates with HIV-1 CA, we used recombinant HIV-1 capsid-nucleocapsid (CA-NC) assemblies to detect binding of either N-terminal or C-terminal POM121 proteins from cell lysates. After incubation of the HIV-1 CA-NC complexes with POM121 containing cell lysates, the mixtures were ultracentrifuged through a sucrose cushion. Both N-terminal and C-terminal POM121 protein were not detected in the pellet in the absence of added CA-NC complexes. After incubation with HIV-1 CA-NC complexes, the C-terminal POM121 protein showed strong interaction with WT CA-NC complexes. By contrast, the N-terminal POM121 demonstrated significantly reduced binding to WT CA-NC complexes (Fig. 5c). Interestingly, N74D CA-NC complexes bound to C-terminal POM121 as efficiently as WT CA-NC complexes, which is consistent with the infection block observed using TRIM5 fusion proteins.

**Fig. 6:**
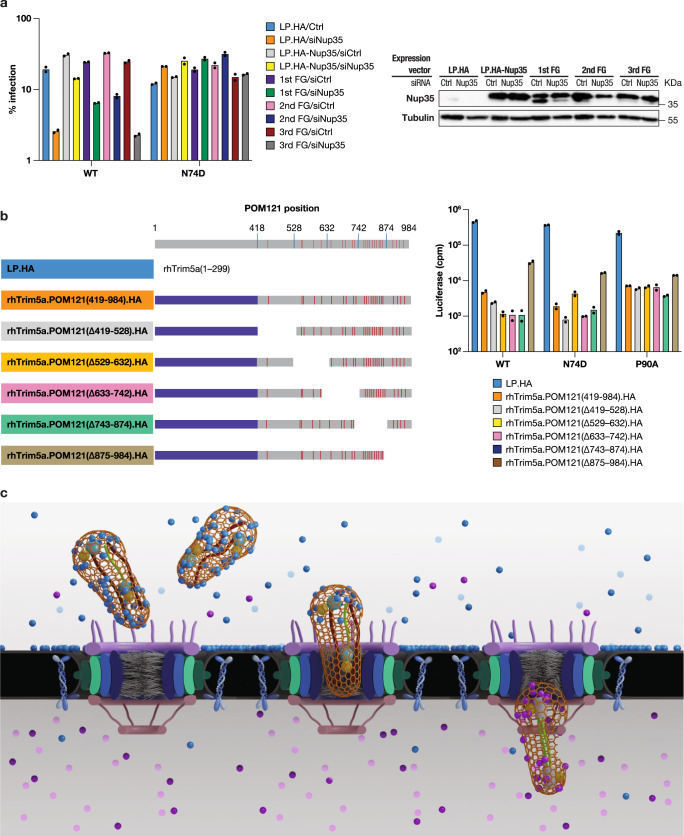
POM121 and Nup35 FG-motif is essential for binding to HIV-1 capsid. **a** HeLa cells stably expressing either wild-type or FG mutant HA-Nup35 lacking 3′-UTR or transduced with control vector (LPCX-HA), were transfected with non-target (NT) siRNA or Nup35 siRNA targeting 3′-UTR region for 48 h, then infected with VSV-G-pseudotyped viruses. *n* = 2 technical replicates, representative of three independent experiments. Western blot analysis confirmed Nup35 restoration. 1st FG, FG179AA; 2nd FG, FG232AA; 3rd FG, FG324AA. **b** Mapping of POM121 C-terminal region required for Trim5a.POM121 restriction. Left panel, schematic representation of systemic deletion constructs of POM121. Blue lines represent boundaries of each constructs, while red lines denote the locations of FG motifs. Right panel, WT HIV-1 or CA-mutant HIV-1 infection of vector or TRIMa.POM121-expressing HeLa cells. *n* = 2 technical replicates, representative of three independent experiments. **c** CypA determines the HIV-1 nuclear import pathway. Premature CPSF6 interaction with HIV-1 impairs CA interaction with FG-Nups at the NPC. CypA binding by HIV-1, however, facilitates use of Nup35, Nup153, and POM121 during infection. Subsequent CPSF6 binding may enhance release from the NPC. We propose that the HIV-1 core, comprised of multimeric CA in association with the viral nucleic acid and enzymatic proteins, directly functions as a NTR and exploits successive FG interactions to achieve transfer through the NPC but these interactions are regulated in time and space by CypA and CPSF6. CypA, azure dot; CPSF6, lilac dot. Source data are provided as a Source Data file.

We used the same assay to investigate binding of Nup35 to HIV-1. HA-tagged Nup35 obtained from cell lysates strongly bound to CA-NC complexes (Supplementary Fig. 9b). Notably, mutation of the C-terminal FG-dipeptide exhibited an attenuated binding ability to CA-NC complexes. To examine the role of Nup35 FG dipeptides during infection, we depleted cells of endogenous Nup35 and ectopically expressed Nup35 with or without FG mutations (Fig. 6a). Expression of the first or second mutant FG construct rescued WT HIV-1 infectivity to 50% of control level. In comparison, expression of the third FG mutant did not restore WT infection in cells depleted of endogenous Nup35. Under these experimental conditions, N74D HIV-1 was insensitive to the different Nup35 manipulations.

C-terminal POM121 contains 25 FG repeats (Fig. 6b). In order to further identify which domains are responsible for HIV-1 inhibition, we generated cell lines stably expressing rhTRIM5 fusion proteins with sequential deletions of C-terminal POM121 (Fig. 6b and Supplementary Fig. 9c). Deletion of residues 419 to 528 resulted in 192-fold inhibition of HIV-1 infection, which was greater than the C-terminal full-length construct. Similarly, fusion constructs with deletions encompassing POM121 residues 529 to 874 exhibited potent HIV-1 inhibition. By contrast, deletion of C-terminal residues 875 to 984 partially lost the ability to inhibit HIV-1 infection (Fig. 6b). Control virus MLV infection was not affected by any of deletion constructs (Supplementary Fig. 9c).

## Discussion

Here we demonstrate the dependence of HIV-1 on Nup35 and POM121 for nuclear entry. As has been observed for Nup153 and Nup358, HIV-1 CA determines sensitivity to loss of Nup35 or POM121. Extending prior work, soluble CA-interacting factors also regulate the necessity for the nucleoporins. Unlike smaller cargo that interacts with cellular NTRs to achieve nuclear entry, we propose that the HIV-1 core, comprised of multimeric CA in association with the viral nucleic acid and enzymatic proteins, directly functions as a macromolecular NTR that encapsulates the cargo and negotiates multiple nucleoporin interactions to achieve transfer through the NPC, through a pathway determined by soluble host factor binding in the cytoplasm. Like cellular NTRs, CA selectively interacts with FG dipeptides^3,6,16^. In contrast to cellular NTRs, HIV-1 can enter the nucleus of nonproliferating cytokine-stimulated T cells and macrophages with the requirement of RanGTPase being unclear^29,30^. Given that the host cell cytoplasm contains sensors to detect and interfere with microbial pathogens, slipping into the nucleus quickly is advantageous to the virus. Indeed, HIV-1 cores have been detected in transition through NPCs^31,32^, and CA structures persist in the nucleus after entry^11,26^.

Although only 24 kilodaltons in size as a monomer, the hundreds of CA molecules present in the hexameric and pentameric complexes composing the cytoplasmic HIV-1 core likely enable a distribution of protein–protein interactions with host factors based on local concentrations and relative binding affinities. Human host factors present in the cytoplasm known to interact with CA include CPSF6, CypA, and MxB. MxB is thought to be enriched proximal to NPCs after interferon activation of cells^33,34^. Indeed in a rigorous study by Kane and colleagues, HIV-1 restriction by MxB was found to be dependent on specific series of Nups, representing subcomplexes as well as a cohort of proteins in different subcomplexes identifying distinct pathways^15^. This work further substantiated the flexibility of HIV-1 nuclear entry against a backdrop of heterogeneity in NPC composition.

In contrast to MX2, CPSF6 and CypA are present at high steady levels in cells, with CPSF6 predominantly enriched in the nucleus and CypA abundant in the cytoplasm, especially in proximity to the nuclear membrane. The ability of the HIV-1 core to bind multiple factors has led to increasing scrutiny of the choreography of where and when these factors are engaged, whether they are simultaneously bound, where they access different interfaces of the core, and whether they compete for common interfaces. The end goal of the virus, in the different cell types that it infects in vivo, is to access the nucleus to establish a provirus. The concentration and distribution difference between CPSF6 and CypA are likely exploited by the virus, to ensure binding surfaces on CA are available for interaction with specific Nups, and to utilize CPSF6 in the nucleus to access gene-rich regions of chromatin. Prior studies have indicated that it is detrimental to the virus to engage these factors out of order. Enrichment of CPSF6 in the cell cytoplasm blocks HIV-1 nuclear entry presumably by preventing Nup interactions with the virus^12,35^.

The role of CypA in HIV-1 infection has been a long-standing puzzle^36,37^. It was one of the first host factors identified to interact with the Gag protein of an animal retrovirus^38^. Although it had been mapped to exert its effect on early replication steps prior to nuclear entry^39,40^, the precise mechanism was unclear. Studies with Nup153 and Nup358 suggested that CypA could be affecting HIV-1 interactions with the NPC^7,27^. It is possible that the essential role that these Nups play in the transport of all cargo limits analysis after they are depleted from cells. In the current study, the effect of CypA on HIV-1 dependence of Nup35 or POM121 was dramatic. While HIV-1 was blocked for infection in Nup35 or POM121 knockdown cells, after treatment with CsA, HIV-1 enters the nucleus and infection is restored to levels of control cells. Studies by others reveal that CypA affects the kinetics of HIV-1 nuclear entry^41^. Collectively, these findings highlight a critical role of CypA in directing HIV-1 to the NPC and potentially dictating the entry pathway. In cells where Nup35 or POM121 supported nuclear entry is diminished, CypA is counterproductive and directs HIV-1 to a nonproductive pathway.

Nup35 is a member of the Nup93 subcomplex which encircles the central channel Nup62 subcomplex comprised entirely of FG-Nups. Nup35 itself has three FG dipeptides, but it is unknown if these are accessible to cargo. Our in vitro CA-NC binding study reveals that the C-terminal region of Nup35 binds to HIV-1 capsid and indicates that the 3^rd^ FG in this region contributes to the interaction. Further examination is necessary to understand how and where Nup35 interacts with HIV-1 in living cells. Similarly, the C-terminal region of POM121 containing a prion-like LCR strongly binds to HIV-1 capsid. Given the demonstrated interaction of FG dipeptides in CPSF6, Nup153, and Sec24C with the N74 pocket of HIV-1 CA^6,16,28,42^, it is tempting to speculate that this interface of HIV-1 negotiates a multitude of interactions by the core during passage through the NPC. To a large extent, Nup35 and POM121 knockdowns phenocopied Nup153 knockdowns with regards to CA-dependent effects on HIV-1 infection. Similar to Nup153 and CPSF6, TRIM5 fusions to the FG-rich portion of POM121 inhibit HIV-1 infection. Our data indicate that POM121 interaction with CA appears to support HIV-1 infection. Interestingly, the POM121 FG domain also binds to HIV-1 Vpr^43^. POM121 is also known to interact with Sun1^44^. Anchored to KASH proteins, Sun1 and Sun2 multimers can form a linker of nucleoskeleton and cytoskeleton (LINC) complexes which span the nuclear envelope^45^. While depletion of POM121 had a more profound effect on WT HIV-1 infection, binding assays indicated interaction with N74D HIV-1 as well. It is possible that the nuclear entry route used by N74D HIV-1 is not dependent or in some way escapes POM121 interaction in cells used in this study. Elucidating the precise choreography of HIV-1 interactions at the nuclear membrane and during passage through the NPC is critical to understanding the successive contributions of each of these factors. The development of an in vitro transport system with purified HIV-1 cores and isolated human nuclei will be essential to help interrogate these steps.

Our prior work pointed to the existence of distinct routes of nuclear entry available to HIV-1^12^, which split along an axis of WT vs N74D HIV-1 CA exhibiting greater dependence on Nup153 vs Nup155, respectively. We find here that this switch in pathway use is governed in part by CypA. N74D HIV-1 exhibits great sensitivity to the loss of CypA with an infection block in the cytoplasm^19^. When Nup155 loss is coupled with CypA depletion in experiments presented here, a greater than 50-fold decrease in N74D HIV-1 infectivity is observed. Moreover, WT HIV-1 also becomes more dependent on the Nup155-import pathway in CypA-depleted cells. For WT HIV-1, a loss of CypA binding diminishes access to the Nup153-import pathway. For N74D HIV-1, the N74D mutation of CA precludes Nup153 utilization.

Despite WT and N74D HIV-1 exhibiting differences in their use of the NPC, there are common themes. Removal of Nup62 subcomplex members (Nup54, Nup58, and Nup62), which are FG-Nups that form a meshwork in the central channel of the pore restricting the flow of cargo larger than 5 nm in diameter, generally elevated both WT and N74D HIV-1 infection. While the loss of Nup54 and Nup58 elevated infection of WT HIV-1, N74D HIV-1, and P90A HIV-1, removal of Nup62 had a larger positive effect on WT HIV-1 infection. Knockdown of Nup214, an FG-Nup that also is thought to regulate the flow of cargo, also had a greater positive effect on WT HIV-1 infection. FG-Nups are thought to play a dual role in nucleocytoplasmic transport. They act as barriers to the majority of macromolecules, but at the same time, can transiently interact with soluble NTRs and mediate their translocation across the NPC. Due to the limitations of conventional structural biology approaches on intrinsically disordered proteins, determining the precise role of FG-Nups has been a substantial challenge. These data and results from others suggest that soluble factors including CPSF6, CypA, and MX2, can help or hinder presentation of the viral core to the NPC. Once engaged with the NPC, multiple CA hexamers interacting with FG-Nups are likely to have a profound effect on the intrinsically disordered sieve by partitioning the hydrogel and inducing their clustering along the channel walls enabling the virus to migrate through the inner channel (Fig. 6c).

It has become increasingly clear that HIV-1 CA is a key viral determinant to essential replication steps in the cytoplasm, at the nuclear membrane, and within the nucleus^46^—a choreography that is profoundly sensitive to disruption by small molecule inhibitors targeting CA, such as GS-CA1, PF-74, and BI-2^47–50^. Collectively, our work and that of others support a model in the CA core highjacks cytoplasmic transport machinery, such as Sec24C, and then functions as a NTR which binds to different FG motifs present in FG-Nups or CPSF6 in the nuclear channel enabling HIV-1 transport into the nucleus. The transposon Tf1 is similarly hypothesized to use an assembled Gag particle as a multimeric NTR, particularly in interacting with the FG-rich yeast Nup124p during nuclear entry^51^. With regards to animal cell biology, HIV-1 provides an attractive model to interrogate the mechanism of nuclear entry by large macromolecular entities.

## Methods

### Plasmids

pNL4-3-Luc-E-R+ (HIV-1 vpr-positive, env-deleted, encoding firefly luciferase in place of nef) or pHIV-RFP (HIV-1 vpr-negative, env-deleted, encoding RFP in place of nef) were used in transfections with pL-VSV-G to generate VSV-G-pseudotyped HIV-1 vectors, and the MLV-based retroviral vector pMX-RFP was used to generate Moloney-based virus in co-transfections with pJK3, pL-VSV-G, and pCMV-Tat. Vectors for FIV^52^ and SIV_MAC239_^53^ have been described previously.

cDNA encoding human Nup35 was amplified from MGC Human Nup35 sequence-verified cDNA (Accession number BC047029, GE Healthcare, Dharmacon), using primers containing a Kozak sequence and an N-terminal HA tag, and inserted into pLPCX-MCS between the EcoRI and NotI sites. HIV-1 CA mutants, phenylalanine-glycine to alanine Nup35 mutants, HA-Nup35-^179^FG^180^ /AA, HA-Nup35-^232^FG^233^/AA, HA-Nup35-^234^FG^235^/AA, and gRNA resistant mutant, HA-Nup35-res were generated by using the QuikChange II XL site-directed mutagenesis kit (Agilent Technologies).

To generate Trim-fusion constructs, rhesus Trim5a (1-299) were amplified using forward primer containing BglII and Kozak sequence, reverse primer containing the (GGGGS) linker and EcoRI or NotI, then inserted into pLPCX-MCS. Then the N-terminal (1-438) or C-terminal (439-984) domain of human POM121, which were amplified from MGC Human POM121 Sequence-Verified cDNA (Accession number BC008794, GE Healthcare, Dharmacon), using primers containing a C-terminal HA tag, were inserted into pLPCX-rhTrim5a (1-299) between EcoRI and NotI or NotI and ClaI respectively. All coding sequences were confirmed by DNA sequencing.

### Cells and culture conditions

HEK293T and HeLa cell lines were maintained in DMEM supplemented with 10% FBS. HeLa cells were growth arrested by incubation in complete medium containing 2 μg/ml aphidicolin (Sigma) for 24 h and were maintained in this concentration of the drug during the infection. H9, Jurkat, and MT4 cells were grown in RPMI 1640 plus 10% FBS. GHOST cells were maintained in DMEM supplemented with 10% FBS, 500 ug/ml G418, 100 ug/ml hygromycin, and 1 ug/ml puromycin.

To generate stable Nup35-expressing cell lines, HeLa cells were transduced with either wild-type or mutant HA-tagged Nup35, a pLPCX-HA-Nup35 vector containing only the amino acid coding sequence of the human Nup35 cDNA, which excludes the 3′-UTR, and the cells were selected with 1 μg/ml puromycin (Millipore).

### siRNA screen

To identify Nups requirements for HIV-1 infection, 32 human Nups were systematically depleted using two different commercially available siRNA pools (Dharmacon; Sigma). siRNAs were transfected into the HeLa cells at a 50 nM final concentration using RNAiMAX (ThermoFisher Scientific), according to the manufacturer’s instructions. After 2 days, the cells were re-seeded into 24-well plates and infected with VSV-G-pseudotyped RFP reporter viruses at an MOI of 0.5 in the presence of 5 ug/ml polybrene. After an additional 48 h incubation, cells were trypsinized and the percentage of RFP-positive cells was scored by flow cytometry (FACSCalibur, BD Biosciences). As a positive control, siRNA SMARTpool against TNPO3 (Dharmacon) was present on each plate. To exclude false positives (e.g., due to toxicity), WT HIV-1, N74D HIV-1, and P90A HIV-1 infections were performed in parallel to Moloney murine leukemia virus (MLV) infection on the Nup-depleted cells. The first round of the screens was performed using siRNA from Sigma. Those genes that showed a phenotype in the first round were confirmed by Dharmacon siRNA. Therefore, two-thirds of the genes were screened using both Dharmacon on-target siRNA SMARTpool and Sigma MISSION esiRNA. The screening was repeated in at least three independent experiments.

### Viral production

To produce HIV-1 particles, pNL4-3-Luc-E-R+ or HIV-RFP (wild-type and capsid mutants: N57A, Q63/67 A, K70A, N74D, P90A, A92E, G94D, and T107A) was co-transfected with a VSV-G expression vector at a ratio of 3:1 using Hilymax (Dojindo Molecular Technologies). The medium was replaced after overnight incubation and viral supernatants were collected at 48 h post-transfection. Viral supernatants were filtered and their infectivity was determined by using HeLa or GHOST target cells.

MLV stocks were obtained by co-transfection of pLPCX, pJK3, pL-VSV-G, and pCMV-Tat at a ratio of 4:2:1:0.3, respectively, with Hilymax. The medium was replaced one day after overnight incubation and viruses were harvested at 48 h after transfection, passed through a 0.45-μm filter, and used directly to transduce target cells.

### Viral infection

For single-cycle infectivity assays, HeLa cells were plated in 24-well plates at 5 × 10^4^ cells per well, and infected with single or serial-dilutions of VSV-G-pseudotyped HIV-1, SIVmac_239_, FIV, or MLV in the presence of 5 ug/ml polybrene. In some experiments, 2.5 μM CsA (Bedford laboratories) was added to the culture media at the time of infection. At 48 h post-infection, cells were washed, and the infection was analyzed by examining the percentage of RFP or GFP expressing cells using flow cytometry (FACSCalibur, BD Biosciences), or by measuring firefly luciferase activity (Wallac 1450 MicroBeta Trilux Liquid Scintillation Counter & Lumi). Flow cytometry analysis was performed with BD CellQuest Pro version 6.0 (BD).

For microscopy experiments, 2.5 × 10^4^ HeLa cells were grown overnight on glass slides in a 24-well plate. The next day, cells were infected with WT, P90A, or A92E pNL4-3-Luc-E-R+ at an MOI of 50. At 12 h post-infection, cells were washed three times with PBS, fixed, permeabilized, and stained with antibodies.

For analysis of HIV-1 reverse transcription products, HeLa cells were seeded at 2 × 10^5^ cells per well in 6-well plates and infected with VSV-G-pseudotyped wild-type HIV-1 or capsid mutant virus (MOI of 1) in the presence or absence of 2.5 μM cyclosporine A. For the negative control, a reverse transcription inhibitor (EFV 150 nM) was added at the time of infection. The phenotype infection assay was performed in parallel in 24-well plates.

### RNA interference

Three ON-TARGETplus siRNAs directed against human Nup35 were purchased from Dharmacon: siRNA 1, 5′-CUGCUGGUUCCUUCGGUUA-3′; siRNA 2, 5′-AGAUAAAAGUGGCGCUCCA-3′; siRNA 3, 5′-AGUUAUUUCUACC GACACA-3′. HeLa cells were plated at 1.5 × 10^5^ cells per well in 6-well plates and transfected the next day with a final concentration of 40 nM siRNA targeting Nup35 or non-targeting control siRNA (siCONTROL non-targeting siRNA, Dharmacon), using RNAiMAX (ThermoFisher Scientific) according to the manufacturer’s instructions. To generate stable Nup35 knockdown cells, a lentiviral vector and the following GIPZ Lentiviral (GE Healthcare, Dharmacon) shRNAs against hNup35 were used in this study:

GIPZ Lentiviral Human Nup35 shRNA:

V3LHS_364366, TGTCTGTCAGAAATAACCT

V3LHS_380787, TATGAGCTGGTACAACTGG

V3LHS_380788, TGGTTCAGATCCTAACGCG

Lentiviral vector stocks were produced by co-transfection of HEK293T cells with packaging plasmid ΔR8.2, MISSION shRNA or GIPZ Lentiviral shRNA, and pL-VSV-G at a ratio of 1:1:0.5, the culture medium was replaced at 12 h, and viral supernatants were collected and filtered at 48 h. HeLa cells were transduced with the filtered supernatant and selected in 2 ug/ml puromycin.

#### CRISPR-Cas9 knockout

To generate a CRISPR-Cas9 single guide RNA (sgRNA) expression vector, oligonucleotides were annealed and inserted into the pX330 (Addgene plasmid #42230). The following sequence targeting coding regions at the 5′ end of Nup35 was used: 5′-GAAGGGCCACTAATTGATCG-3′. Non-targeting sgRNA was used as control, 5′-CGCTTCCGCGGCCCGTTCAA-3′. HeLa knockout cells were generated by transiently transfecting pX330 into the cells. Single clones were obtained by limiting dilution and screened by western blotting and phenotype assay and the candidate clones were verified by sequencing of genomic DNA using the following primers: 5′-cgGAATTCACATCTCCAAAGCCAGGAGTTA-3′ and 5′-taAAGCTTTGTATTTTATGTGTGGCCCAAG-3′ (target exon3).

### RNAi-resistant mutant generation and phenotype rescue

To generate RNAi-resistant variants of genes, the exogenous ORF transcript lacking the 3′-UTR sequence targeted by the siRNA was inserted into the LPCX vector. HeLa cells were then transduced with the filtered supernatant and the cells were selected with 1 μg/ml puromycin (Millipore). For the rescue assay, puromycin-selected cells were plated at 1.5 × 10^5^ cells per well in 6-well plates and transfected the next day with siRNA (40 nM) against human Nup35 3′-UTR region or control siRNA. Two days post-transfection, cells were re-seeded and infected with the HIV-1 or MLV and analyzed by FACS 2 days post-infection.

### Quantification of HIV-1 reverse transcription products by real-time PCR

HeLa cells were transfected with siRNAs as described above. Two days later, cells were seeded at 2 × 10^5^ cells per well in 6-well plates and infected with VSV-G-pseudotyped HIV-1, or capsid mutant virus P90A HIV-1, or A92E HIV-1. The virus was pretreated with 20 U ml^−1^ RNase-free DNase I (Roche) for 1 h at 37 °C. At 2 h post-infection, the cells were washed once with PBS and fresh complete medium was added, cells were then collected at 3, 6, 12 and 24 h after infection. Total DNA was extracted using the QIAamp DNA Blood Mini Kit (Qiagen) and used for real-time PCR to specifically quantify HIV early reverse transcription (RT) products, late RT products, and 2-LTR circle forms. The primer-probe sets and conditions were used as previously described^54^. To normalize the amount of DNA in each PCR assay, the following primer set was used to quantify the copy number of the cellular gene actin: forward, 5′-TCACCCACACTGTGCCCATCTA CGA-3′ and reverse 5′- CAGCGGAACCGCTCATTGCCAATGG-3′. qPCR assays were performed using either Platinum qPCR SuperMix-UDG (Invitrogen) for detecting HIV-1 reverse transcription or iQ SYBR Green Supermix (Bio-Rad) for detecting actin. The reactions were run by using DNA Engine Opticon (MJ Research, BioRad).

### Western blotting

Whole-cell extracts were prepared by lysing cells in RIPA buffer (Sigma-Aldrich), equivalent protein content boiled in SDS sample buffer, resolved by Criterion Tris-HCl Precast Gels (Bio-Rad), and blotted onto PVDF Blotting Membranes (GE Healthcare). Membranes were probed using mouse monoclonal anti-cyclophilin A (Abcam, Cat. No. ab58144, clone 1F4-1B5, 1:3000 dilution), rabbit polyclonal anti-CPSF6 (Novusbio, Cat. No. NB100-61596, 1:3000 dilution), rabbit polyclonal anti-Nup53 (Abcam, Cat. No. ab126993, 1:1000 dilution), rabbit polyclonal anti-Nup53 (Bethyl, Cat. No. A301-781A, 1:2000 dilution), rabbit polyclonal anti-Nup53 (GeneTex, Cat. No. GTX64510, 1:1000 dilution), rabbit polyclonal anti-Nup53 (Novusbio, Cat. No. NB100-93322, 1:1000 dilution), rabbit polyclonal anti-Nup93 (Abcam, Cat. No. ab168805, 1:500 dilution), mouse monoclonal anti-Nup153 (Abcam, Cat. No. ab24700, 1:500 dilution), rabbit polyclonal anti-Nup155 (Abcam, Cat. No. ab73292, 1:500 dilution), rabbit polyclonal anti-Nup188 (Novusbio, Cat. No. NBP1-28748, 1:1000 dilution), rabbit polyclonal anti-Nup205 (Novusbio, Cat. No. NBP1-91247, 1:750 dilution), rabbit polyclonal anti-Nup358 (Abcam, Cat. No. ab64276, 1:1000 dilution), rabbit polyclonal anti-POM121 (GeneTex, Cat. No. GTX102128, 1:500 dilution), mouse monoclonal anti-TNPO3 (MyBioSource, Cat. No. MBS120261, 1:200 dilution), mouse monoclonal anti-HA (Sigma, No. H9658-2ML, 1:1500 dilution), mouse monoclonal anti-tubulin (Sigma, Cat. No. T6074, 1:50,000 dilution), and Human Anti-Human Immunodeficiency Virus Type 1 Neutralizing Serum 1, Heat-inactivated (HIV reagent program, Cat. No. ARP-1984) 1:250 dilution followed by secondary HRP-conjugated anti-mouse (GE Healthcare, Cat. No. NA931V, 1:10,000 dilution) or anti-rabbit (GE Healthcare, Cat. No. NA934V, 1:10,000 dilution) antibodies and detected using a Chemidoc XRS+ system (Bio-Rad). Uncropped and unprocessed scans of the most important blots are provided in the Source Data file.

### Immunofluorescence

Cells were fixed with 4% paraformaldehyde (Boston Bioproducts) at room temperature for 10 min, permeabilized with 0.2% Triton X-100 in PBS for 10 min, and then blocked with 3% BSA for 30 min. Cells were incubated with primary antibodies at RT for 1 h, followed by Alexa-Fluor-conjugated secondary antibodies and Hoechst 33342 (Thermo Fisher Scientific) at RT for 30 min. Coverslips were mounted on glass slides with ProLong Gold antifade solutions (Molecular Probes). Mouse monoclonal anti-Cyclophilin A (Abcam, Cat. No. ab58144, clone 1F4-1B5, 1:2000 dilution), rabbit polyclonal anti-CPSF6 (Novusbio, Cat. No. NB100-61596, 1:400 dilution), mouse monoclonal anti-Nuclear Pore Complex (BioLegend, Cat. No. 902907, clone MAb 414, 1:200 dilution), and rabbit polyclonal anti-Lamin B1 (Abcam, Cat. No. ab16048, 1:2000 dilution) were used as primary antibodies. Secondary antibodies included goat anti-rabbit Alexa Fluor 488 (Molecular Probes, Cat. No. A11008, 1:2000 dilution), goat anti-mouse Alexa Fluor 488 (Molecular Probes, Cat. No. A11017, 1:2000 dilution), goat anti-mouse Alexa Fluor 546 (Molecular Probes, Cat. No. A11019, 1:2000 dilution), and goat anti-rabbit Alexa Fluor 555 (Molecular Probes, Cat. No. A21429, 1:2000 dilution).

### Deltavison deconvolve microscopy

Fixed samples were taken in Z-stacks using the Deltavison deconvolve microscope (GE Healthcare) and deconvolved to remove out-of-focus light using the Softworks software (Version 7.0.0, GE Healthcare).

### Super-resolution imaging using structured illumination microscopy (SIM)

HeLa control and Nup35 knockdown cells were fixed and stained with rabbit anti-Nup155 (Abcam) and mouse anti-Nup153 (Abcam) at room temperature for 1 h and incubated with goat anti-rabbit Alexa Fluor 488 (Molecular Probes) and goat anti-mouse Alexa Fluor 546 (Molecular Probes) at room temperature for 1 h. Subsequently, samples were incubated for 20 min at room temperature with directly conjugated rabbit anti-Lamin B1 antibody, which was labeled using Zenon^TM^ Alexa Fluor^TM^ 647 Rabbit IgG Labeling Kit (Zenon) according to the manufacturer’s instructions. 3D SIM imaging was performed on a DeltaVision OMX SR microscope (GE Healthcare) equipped with a 60× 1.40 numerical aperture Plan Apochromat oil immersion objective (Olympus). Samples were mounted with ProLong Gold Mounting Medium (Invitrogen) and image stacks with 15 images per plane (five phases, three angles) and a z-distance of 125 nm were captured and then subjected to a computational reconstruction (softWoRX; Version 6.1, Applied Precision).

### Protein expression and purification

pET-11a vectors were used to express the HIV-1 capsid protein. Point mutations, A14C and E45C were introduced using the QuikChange II site-directed mutagenesis kit (Stratagene) according to the manufacturer’s instructions. All proteins were expressed in *Escherichia coli* one-shoot BL21star^TM^ (DE3) cells (Invitrogen). Briefly, LB medium was inoculated with overnight cultures, which were grown at 30 °C until mid log-phase (*A*_600_, 0.6–0.8). Protein expression was induced with 1 mM isopropyl-β-d-thiogalactopyranoside (IPTG) overnight at 18 °C. Cells were harvested by centrifugation at 5000 × *g* for 10 min at 4 °C, and pellets were stored at −80 °C until purification. Purification of capsid was carried out as follows. Pellets from 2 L of bacteria were lysed by sonication (Qsonica microtip: 4420; *A* = 45; 2 min; 2 s on; 2 s off for 12 cycles), in 40 ml of lysis buffer (50 mM TRIS Ph = 8.50 mM NaCl, 100 mM β-mercaptoethanol and Complete EDTA-free protease inhibitor tablets). Cell debris were removed by centrifugation at 40,000 × *g* for 20 min at 4 °C. Proteins from the supernatant were precipitated by incubation with 1/3 of volume of saturated ammonium sulfate containing 100 mM β-mercaptoethanol for 20 min at 4 °C and centrifugation at 8000 × *g* for 20 min at 4 °C. Precipitated proteins were resuspended in 30 ml of buffer A (25 mM MES pH6.5, 100 mM β-mercaptoethanol) and sonicated 2-3 times (Qsonica microtip: 4420; A = 45; 2 min; 1 s on; 2 s off). The sample was dialyzed 3 times in buffer A (2 h, overnight, 2 h). The sample was sonicated and diluted in 500 ml of buffer A and was chromatographed sequentially on a 5 ml HiTrap^TM^ Q HP column and on a 5 ml HiTrap^TM^ SP FF column (GE Healthcare), both preequilibrated with buffer A. The capsid protein was eluted from HiTrap^TM^ SP FF column using a linear gradient from 0 to 2 M of NaCl. Absorbance at 280 nm was checked to take the eluted fraction that had higher protein levels. Pooled fractions were dialyzed 3 times (2 h, overnight, 2 h) in storage buffer (25 mM MES, 2 M NaCl, 20 mM β-mercaptoethanol). The sample was concentrated using centricons to a concentration of 20 mg/ml and stored at −80 °C.

### Assembly of stabilized HIV-1 capsid tubes

One microliter of monomeric capsid (3 mg/mL or 1 mg/mL) was dialyzed in SnakeSkin dialysis tubing 10,000 MWCO (Thermo Scientific) against a buffer that is high in salt and contains a reducing agent (buffer 1: 50 mM Tris, pH 8, 1 M NaCl, 100 mM β-mercaptoethanol) at 4 °C for 8 h. Subsequently the protein was dialyzed against the same buffer without the reducing agent β-mercaptoethanol (buffer 2: 50 mM Tris, pH 8, 1 M NaCl) at 4 °C for 8 h. The absence of β-mercaptoethanol in the second dialysis allows formation of disulfide bonds between Cysteine 14 and 43 inter-capsid monomers in the hexamer. Finally, the protein is dialyzed against buffer 3 (20 mM Tris, pH 8,0, 40 mM NaCl) at 4 °C for 8 h. Assembled complexes were kept at 4 °C up to 1 month.

### Capsid binding assay

Human HEK293T cells were transfected for 24 h with a plasmid expressing the protein of interest. Cell media was completely removed and cells were lysed in 300 μL of capsid binding buffer (CBB: 10 mM Tris, pH 8,0, 1,5 mM MgCl_2_, 10 mM KCl) by scrapping off the plate. Cells were rotated at 4 °C for 15 min and then centrifuged to remove cellular debris (21,000 × *g*, 15 min, 4 °C). Cell lysates were incubated with stabilized HIV-1 capsid tubes for 1 h at 25 °C. Subsequently, stabilized HIV-1 capsid tubes were washed by pelleting the complexes by centrifugation at 21,000 × *g* for 2 min. Pellets were washed using by resuspension in CBB or PBS. Pellets were resuspended in Laemmli buffer 1X and analyzed by Western blotting using anti-p24 and the indicated antibodies.

### Reporting summary

Further information on research design is available in the Nature Portfolio Reporting Summary linked to this article.

## Supplementary information

Supplementary Information

Reporting Summary

## Data Availability

The authors declare that the data supporting the findings of this study are available within the paper and its supplementary information files. siRNA, shRNA, and gRNA sequences are provided in Materials. Source data are provided in a supplementary document. Source data are provided with this paper.

## Source data

Source Data
