# Supplementary material for: The HIV-1 capsid core is an opportunistic nuclear import receptor
Source: Nat Commun. 2023 Jun 24;14:3782. doi: 10.1038/s41467-023-39146-5 (PMC10290713; doi:10.1038/s41467-023-39146-5)
Supplement: Supplementary file 1 — Supplementary Information [file 41467_2023_39146_MOESM1_ESM.pdf]

Supplementary Information

The HIV-1 capsid core is an opportunistic nuclear import receptor

Xue et al.

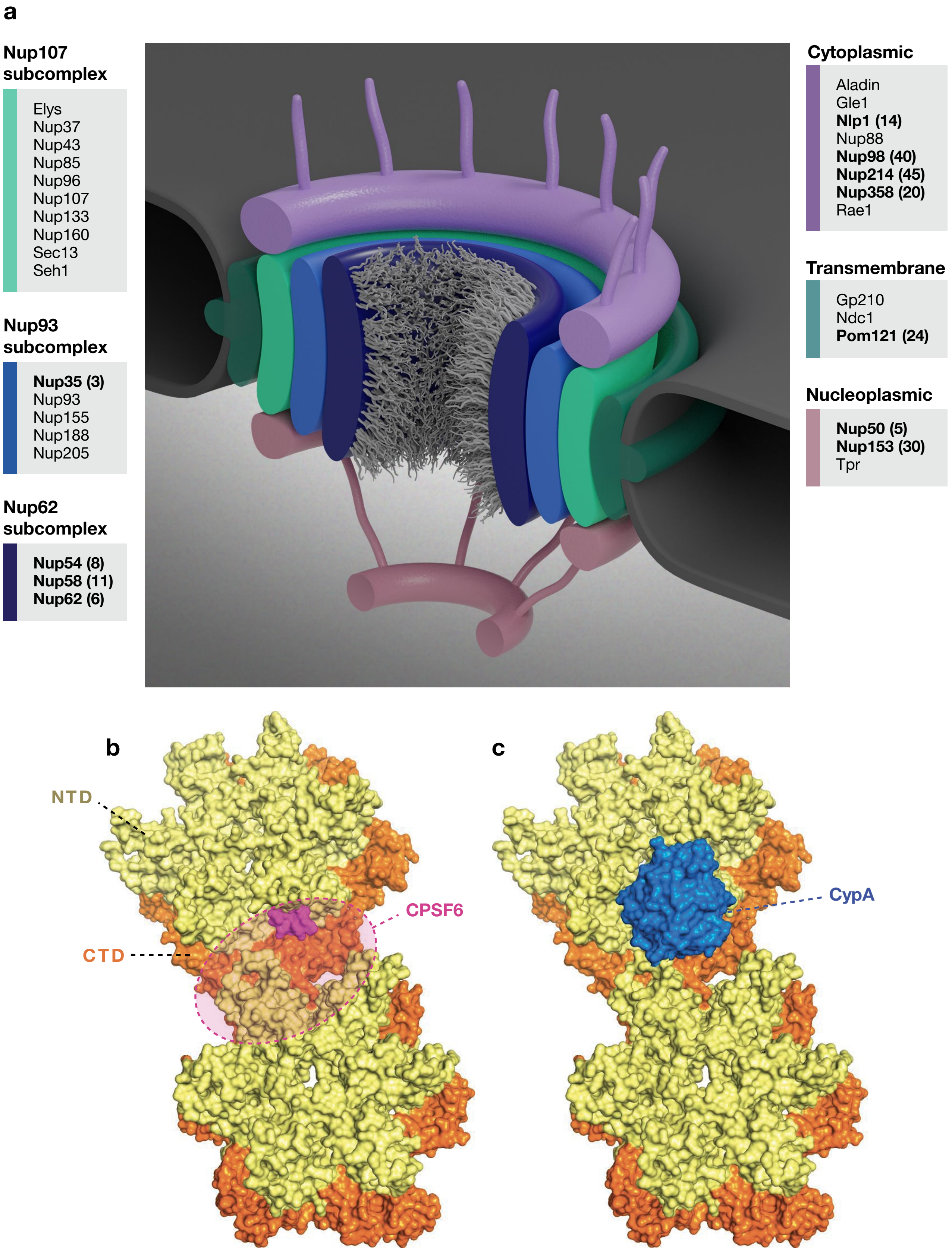

Supplementary Figure 1: Depiction of the nuclear pore complex and HIV-1 CA hexamers. a, Major human NPC subcomplexes. FG-Nups hypothesized to interact with nuclear pore cargo based on yeast or metazoan studies are emboldened, and the numbers of FG repeats are added. **b and c**, CypA and CPSF6 binding regions in the HIV-1 capsid. Surface representation of the structure of two adjacent hexamers from the cryo-EM model of capsid (CA) tubes (PDB ID: 3J34; yellow CANTDS and orange CA_{CTDS}). (**b**) Binding site of a CPSF6 peptide (residues 313–327; magenta) based on crystal structures PDB ID: 4U0A and 4WYM. (**c**) Binding site of CypA based on cryo-EM model PDB ID: 5FJB; blue.

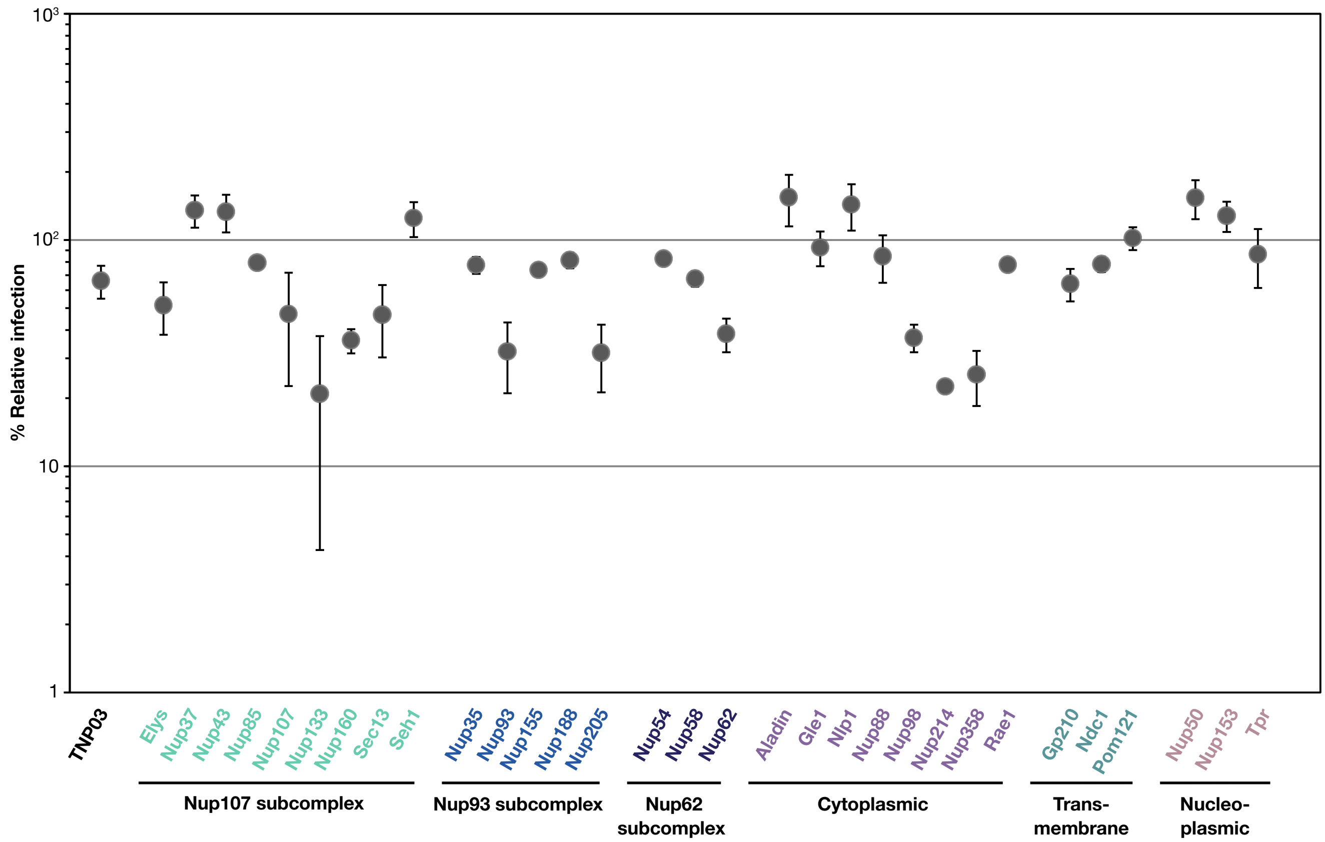

Supplementary Figure 2: Effect of nucleoporin knockdown on MLV infection. Wild-type (WT) or CA-mutant HIV-1-RFP reporter virus infection of control or target siRNA transfected HeLa cells. This analysis was performed in parallel the experiments shown in Fig. 1a. Percent relative infection is mean ± s.d., n=2–5 for nucleoporins, n=15 for TNPO3 from biologically independent experiments. MLV, murine leukemia virus.

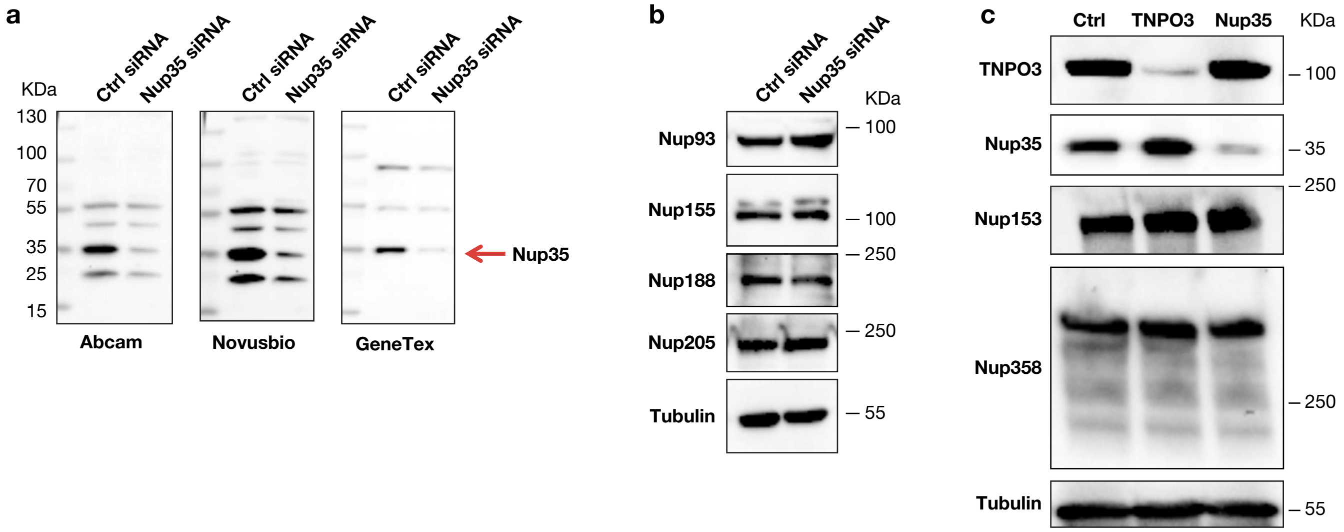

Supplementary Figure 3: Nup35 knockdown does not affect levels of other nucleoporins. Nup35 knockdown does not affect levels of other proteins in the Nup93 subcomplex. Western blot analysis with anti-Nup35 (**a**) or antibodies against Nup93 subcomplex proteins (**b**) using lysates from Nup35-knockdown HeLa cells. **c**, Nup153 or Nup358 levels are unchanged in Nup35 knockdown cells. Western blot analysis showing reactivity to anti-Nup35 or antibodies against TNPO3, Nup153, and Nup358 using Nup35 or TNPO3 knockdown HeLa cell lysates.

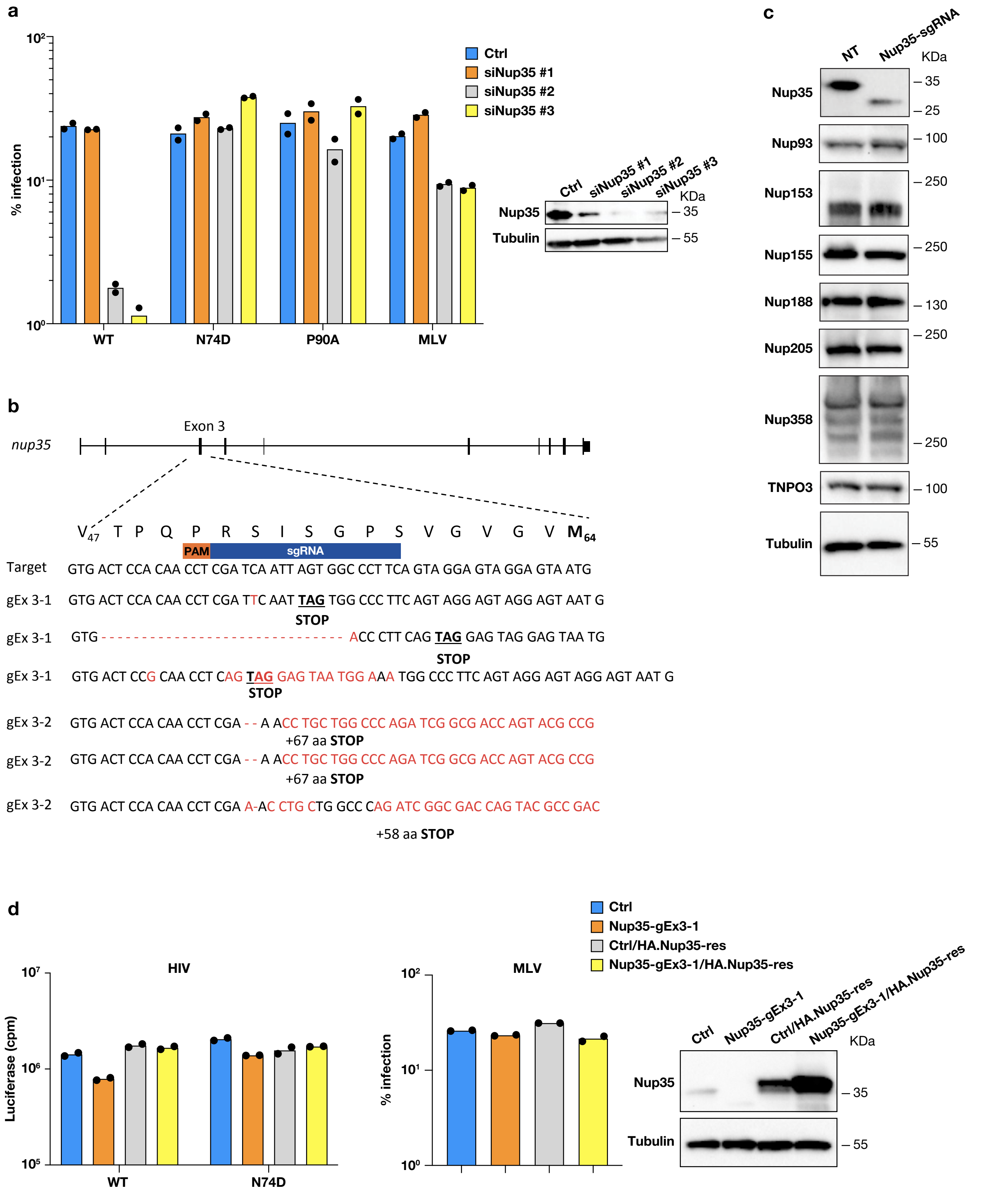

Supplementary Figure 4: Generation of knockout HeLa cells. **a**, Different siRNAs targeting Nup35 impair HIV-1 infection. WT HIV-1 or CA-mutant HIV-1 or MLV infection of control or three different siRNAs against Nup35 transfected HeLa cells. n=2 technical replicates, representative of three independent experiments. Western blot analysis confirmed Nup35 depletion by three different siRNAs. **b**, Genotyping of *nup35* knockout clonal HeLa cell line. The protospacer adjacent motif (PAM) region is shown in orange, and the CRISPR-Cas9 nuclease target sequence in blue. Gene editing events are indicated in red at the bottom of the wild-type sequence. **c**, Expression of nucleoporins in Nup35 knockout cells. Western blot analysis of Nup93 subcomplex proteins as well as TNPO3, Nup153, and Nup358 from Nup35 knockout HeLa cell lysates. **d**, Two Nup35 knockout HeLa cell clones were transduced with gRNA resistant form of Nup35, then infected with VSV-G-pseudotyped viruses. n=2 technical replicates, representative of two independent experiments. Western blot confirmed Nup35 restoration.

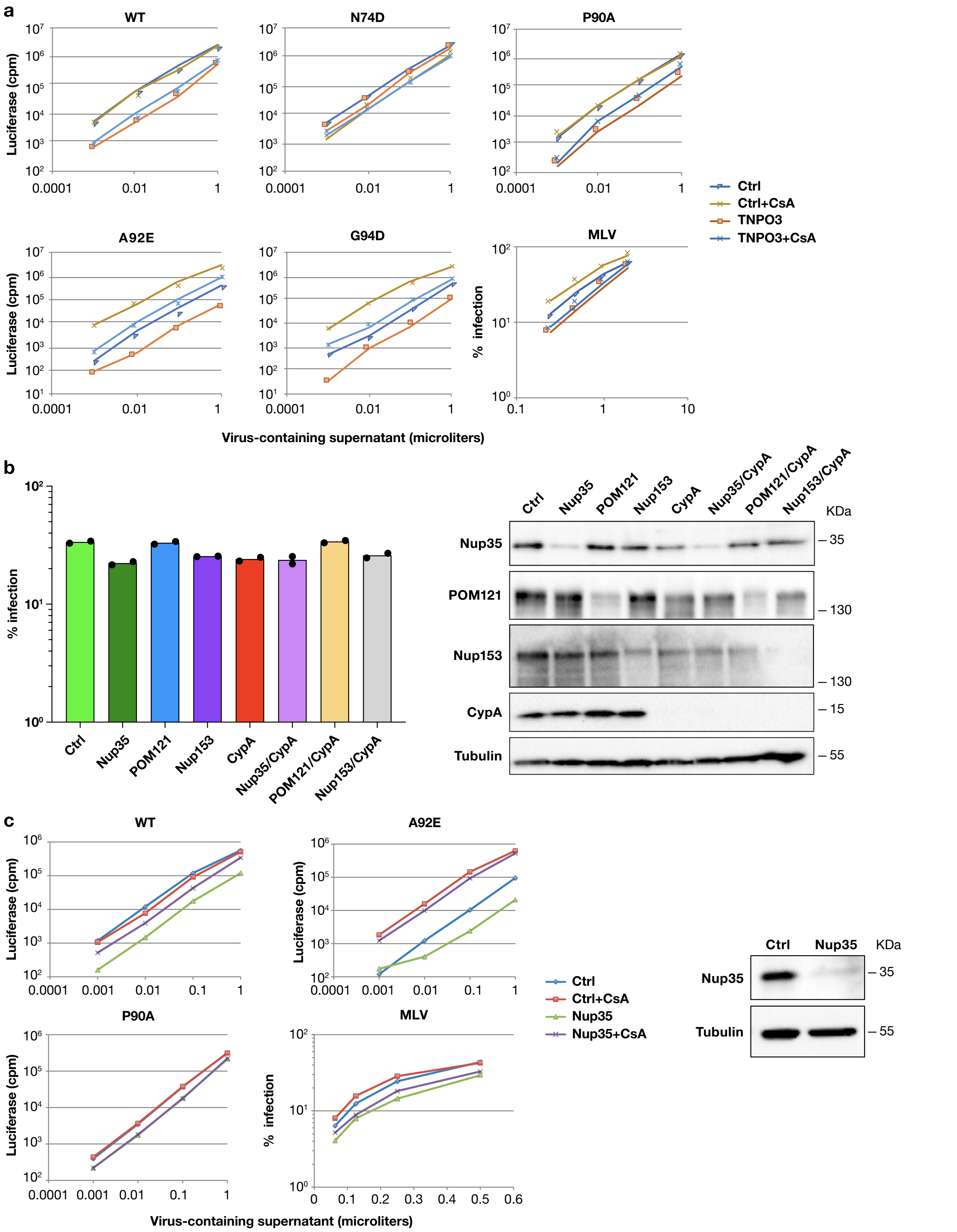

Supplementary Figure 5: a, WT HIV-1 or CA-mutant HIV-1 or MLV infection of control or TNPO3 siRNA transfected HeLa cells in the presence or absence of CsA. n=2 technical replicates, representative of three independent experiments. This analysis was performed in parallel to the experiments shown in Fig. 2c. **b**, Knockdowns of CypA-dependent nucleoporins have minor effects on MLV infection. MLV infection of control or single-knocked down or double-knocked down HeLa cells. This analysis was performed in parallel to the experiments shown in Fig. 2d. n=2 technical replicates, representative of three independent experiments. Western blotting confirmed knockdown efficiency. **c**, WT HIV-1 or CA-mutant HIV-1 or MLV infection of control or Nup35 siRNA transfected HeLa cells in the presence or absence of CsA. n=2–3 technical replicates, representative of two independent experiments. Western blotting confirmed knockdown efficiency. This analysis was performed in parallel the experiments shown in Fig. 3b.

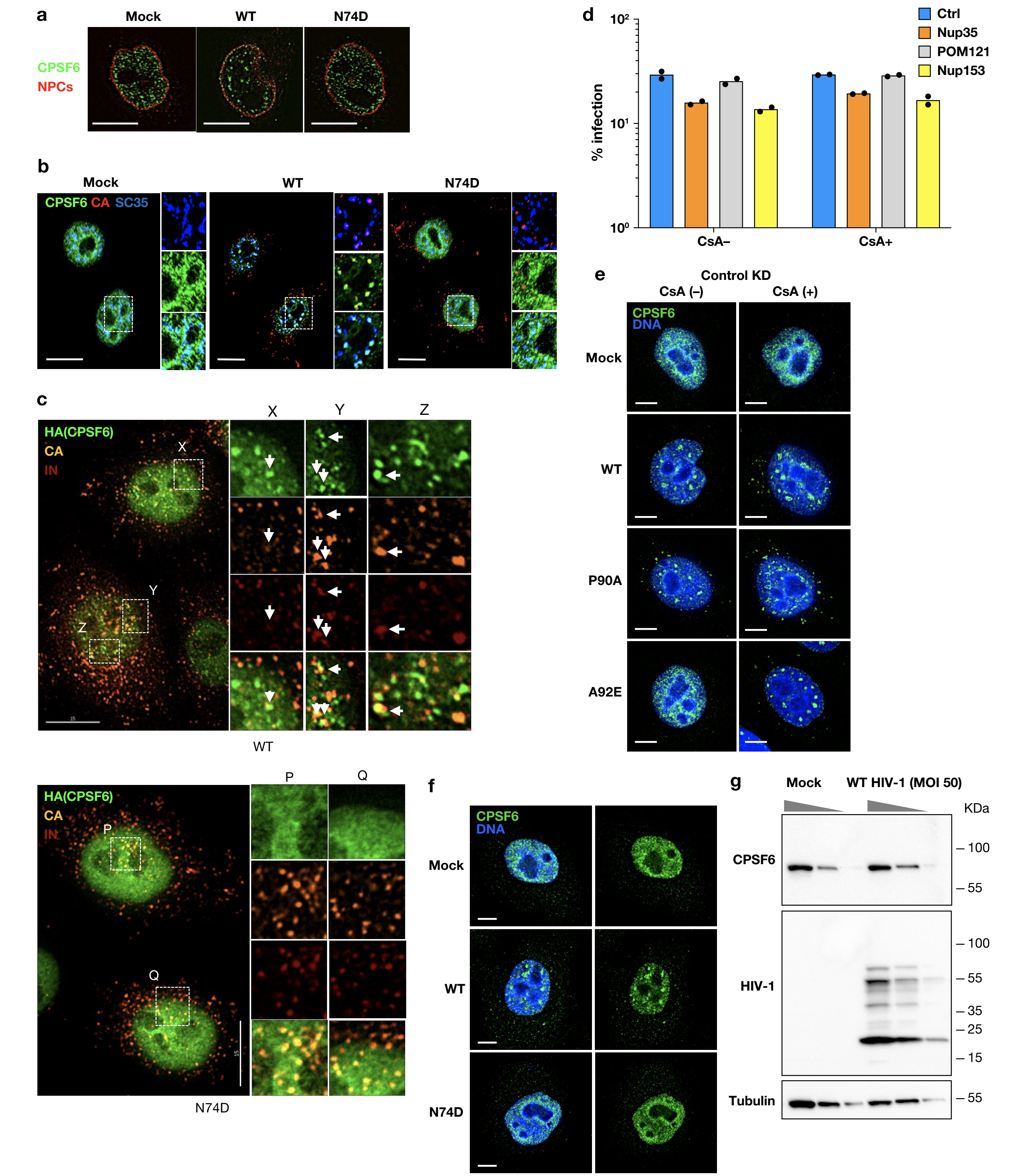

Supplementary Figure 6: a and **b**, WT HIV-1 or N74D HIV-1 infection of HeLa cells. Cells were fixed at 16 hpi and analyzed by super-resolution microscopy in (**a**) and stained for CPSF6, CA, and SC35 and analyzed by deconvolution microscopy in (**b**). The colocalization of CPSF6, CA, and SC35 in WT HIV-1 infected cells were shown in insets. **a and b**, Representative of 40 or 50 cells from two independent experiments, respectively. Scale bars: 10 µm. **c**, WT HIV-1 or N74D HIV-1 infection of CPSF6-expressing HeLa cells. Cells were fixed at 16 hpi, and then stained for HA, CA, and IN. Representative regions of each cell were magnified in insets and the association of CPSF6 with viral CA and IN was indicated by arrows. Representative of 30 cells from two independent experiments. Scale bars: 15 µm. **d**, WT HIV-1 or CA-mutant HIV-1 infection of control or target siRNA transfected HeLa cells in the presence or absence of CsA. n=2 technical replicates, representative of three independent experiments. –, without CsA; +, with CsA. **e**, An enlarged version of Fig. 3d. Representative of 50 cells from two independent experiments. **f**, WT HIV-1 infection of HeLa cells. After 12 h, cells were fixed and stained with antibodies against CPSF6. Representative of 50 cells from two independent experiments. Scale bar, 5 µm. **g**, HIV-1 infection does not affect endogenous CPSF6 levels. HeLa cells were infected with WT HIV-1. After 12 h infection, cells were collected and whole cell lysates were used for western blot analysis. Western blotting images are from two separate trials and two independent virus stocks.

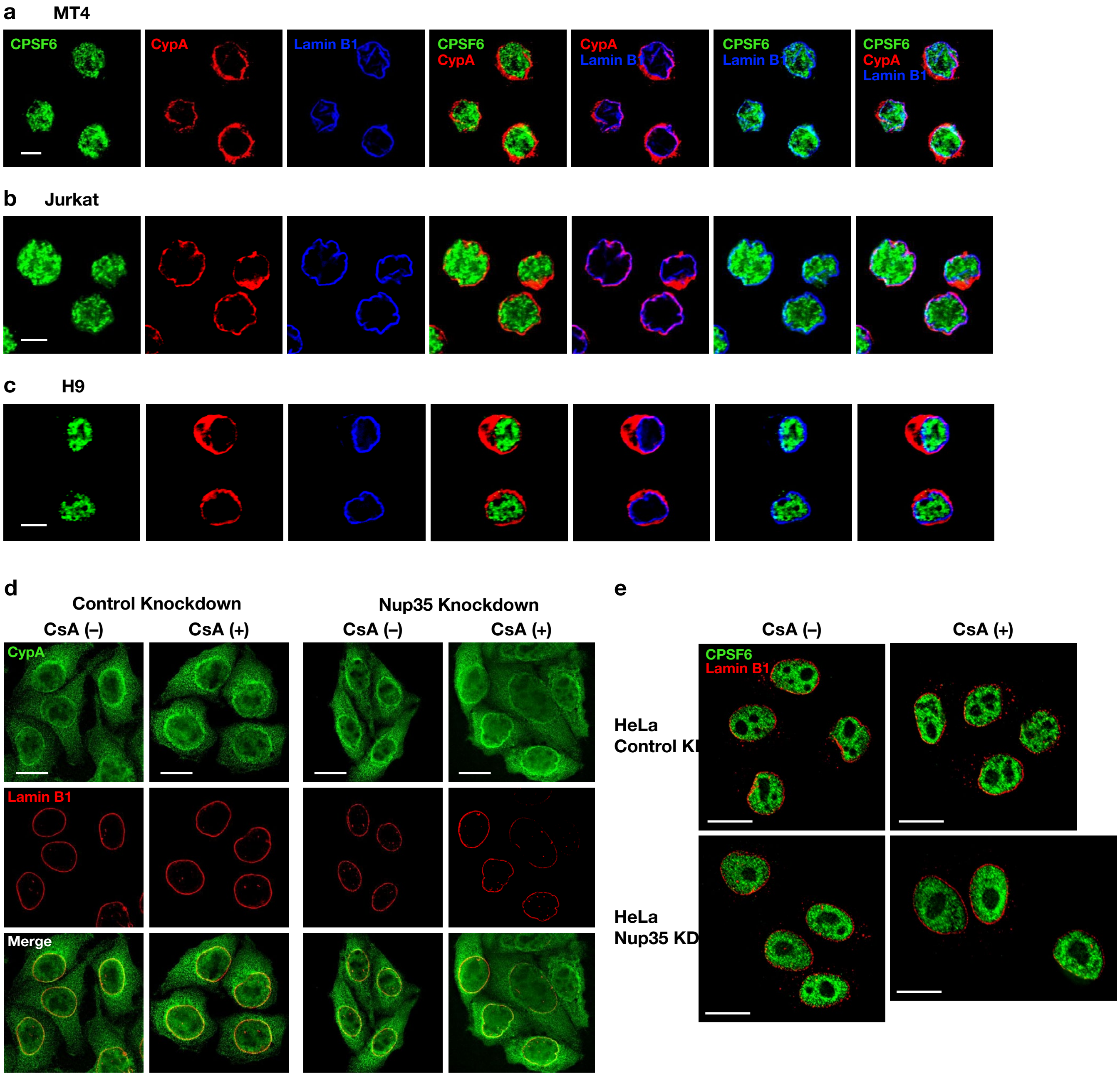

Supplementary Figure 7: Subcellular distribution of soluble factors. a–c, Distribution of CPSF6 and CypA in MT4 (**a**), Jurkat (**b**), and H9 (**c**) cells. Localization of CPSF6 (green), CypA (red), and lamin B1 (blue) in a z-section of immunostained cells imaged by deconvolution microscopy. **a–c**, Representative of 100 cells from two independent experiments. Scale bar, 5 µm. **d**, Nup35 depletion does not alter CypA subcellular localization. Localization of CypA (green) and lamin B1 (red) in a z-section of immunostained HeLa cells imaged by deconvolution microscopy. Representative of 100 cells from two independent experiments. Scale bar, 15 µm. **e**, Nup35 depletion does not alter CPSF6 subcellular localization. Localization of CPSF6 (green) and lamin B1 (red) in a z-section of immunostained HeLa cells imaged by deconvolution microscopy. Representative of 100 cells from two independent experiments. Scale bar, 15 µm.

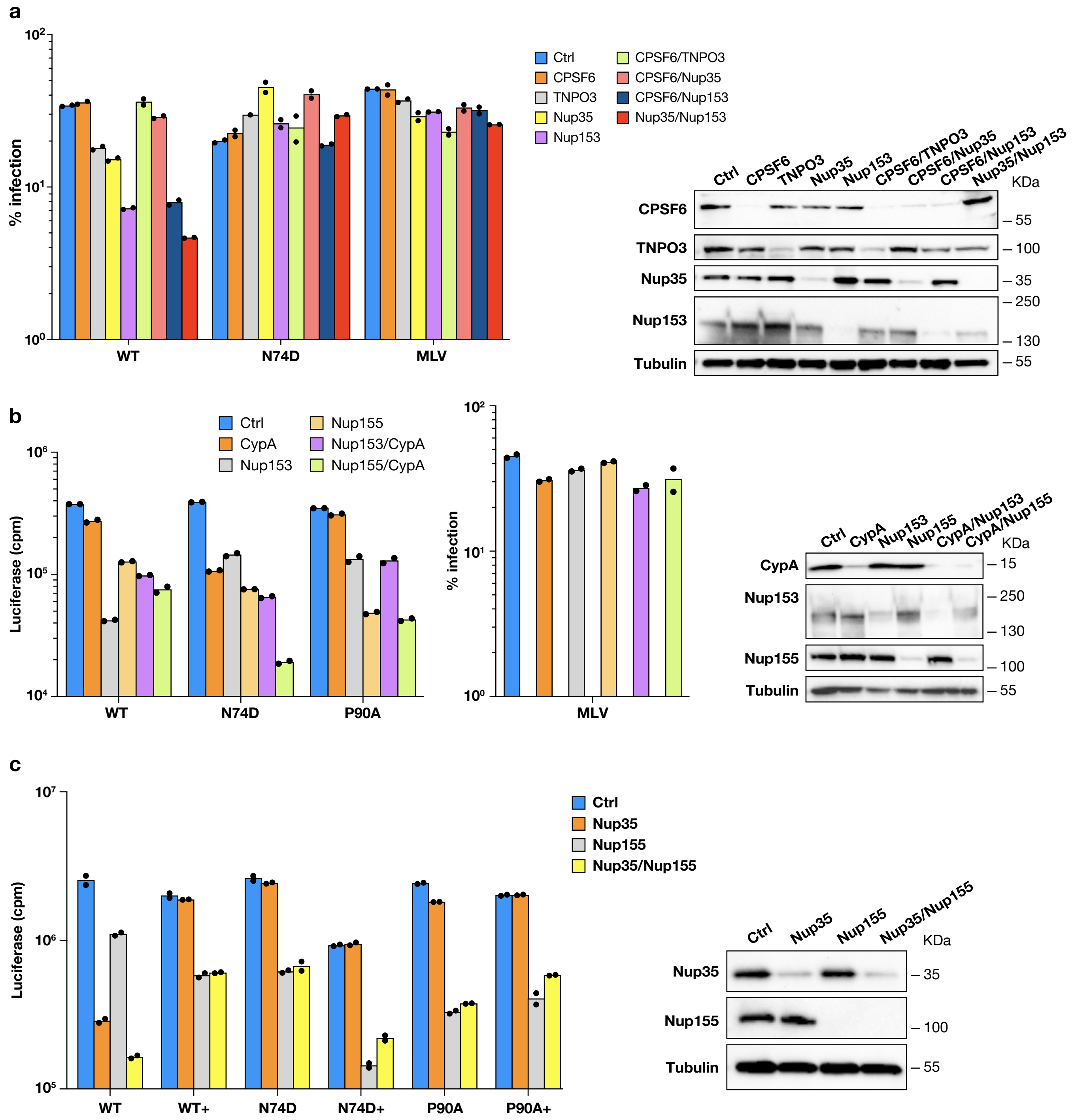

Supplementary Figure 8: a, Distinct contributions of Nup35 and Nup153 to HIV-1 infection. WT HIV-1 or N74D HIV-1 or MLV infection of control or single-knocked down or double-knocked down HeLa cells.n=2 technical replicates, representative of three independent experiments. Western blotting confirmed knockdown efficiency. **b**, CypA determines HIV-1 dependency on Nup153 versus Nup155. WT HIV-1 or CA-mutant HIV-1 or MLV infection of control or single-knocked down or double-knocked down HeLa cells. n=2 technical replicates, representative of three independent experiments. Western blotting confirmed knockdown efficiency. **c**, WT HIV-1 or CA-mutant HIV-1 infection of control or target siRNA transfected HeLa cells in the presence or absence of CsA. n=2 technical replicates, representative of two independent experiments. –, without CsA; +, with CsA. Western blotting confirmed knockdown efficiency.

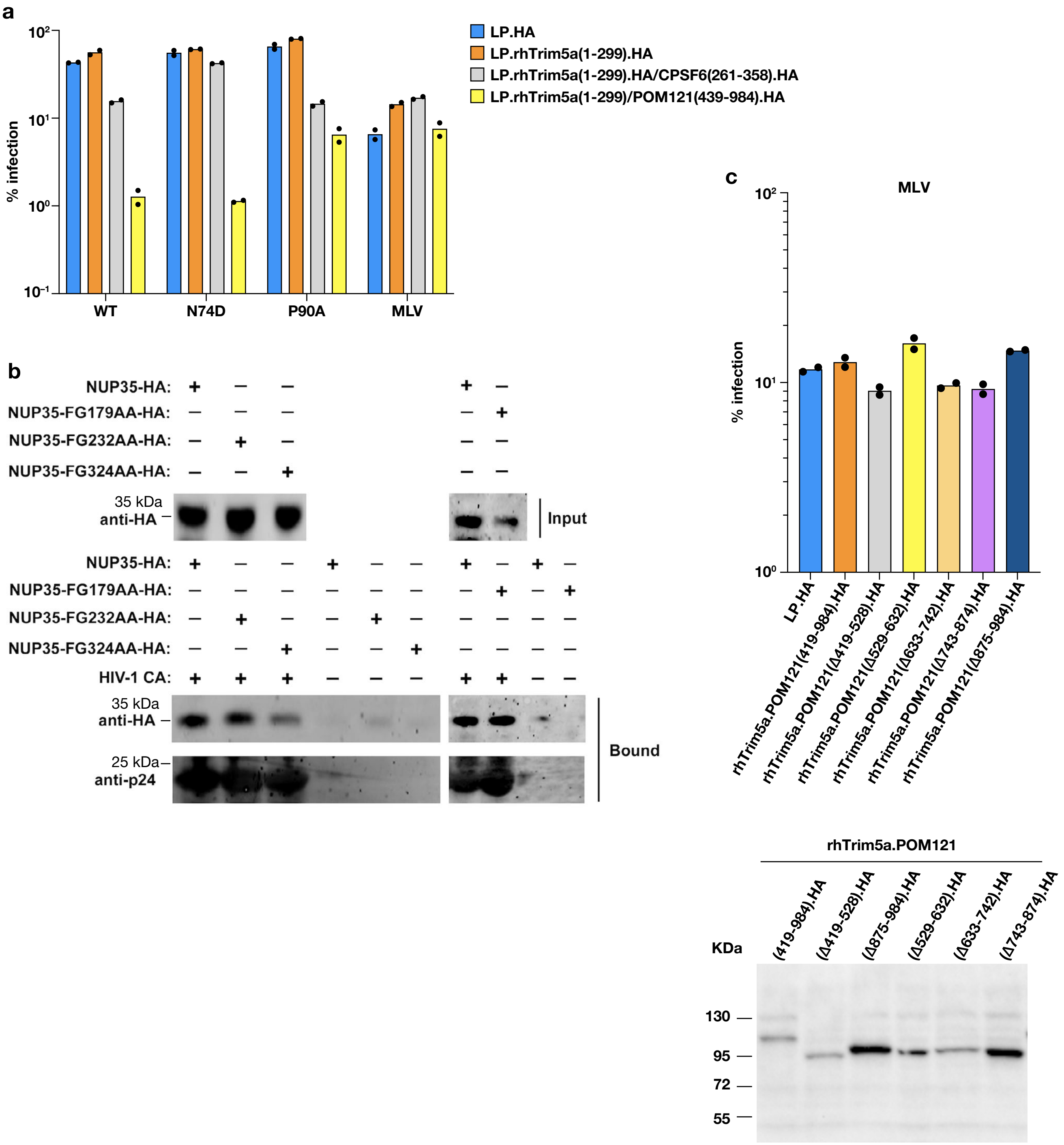

Supplementary Figure 9: a, C-terminal domain of POM121 is required for restriction of HIV-1 infection. WT HIV-1 or CA-mutant HIV-1or MLV infection of vector or rhTRIM-POM121-expressing HeLa cells. n=2 technical replicates, representative of three independent experiments. **b**, Binding of Nup35 to stabilized *in vitro*-assembled HIV-1 CA complexes. Lysates from transfected 293T cells expressing HA-tagged wild type or mutant Nup35 proteins (Input) were incubated with HIV-1 CA complexes for 1 hour. Capsid complexes were washed two times using capsid binding buffer. Samples were analyzed by Western blotting using anti-HA and anti-p24 antibodies (Bound). Similar results were obtained in at least 3 independent experiments. **c**, WT HIV-1 or CA-mutant HIV-1 infection of vector or rhTRIM-POM121-expressing HeLa cells. n=2 technical replicates, representative of three independent experiments. This analysis was performed in parallel to the experiments shown in Fig. 6b.

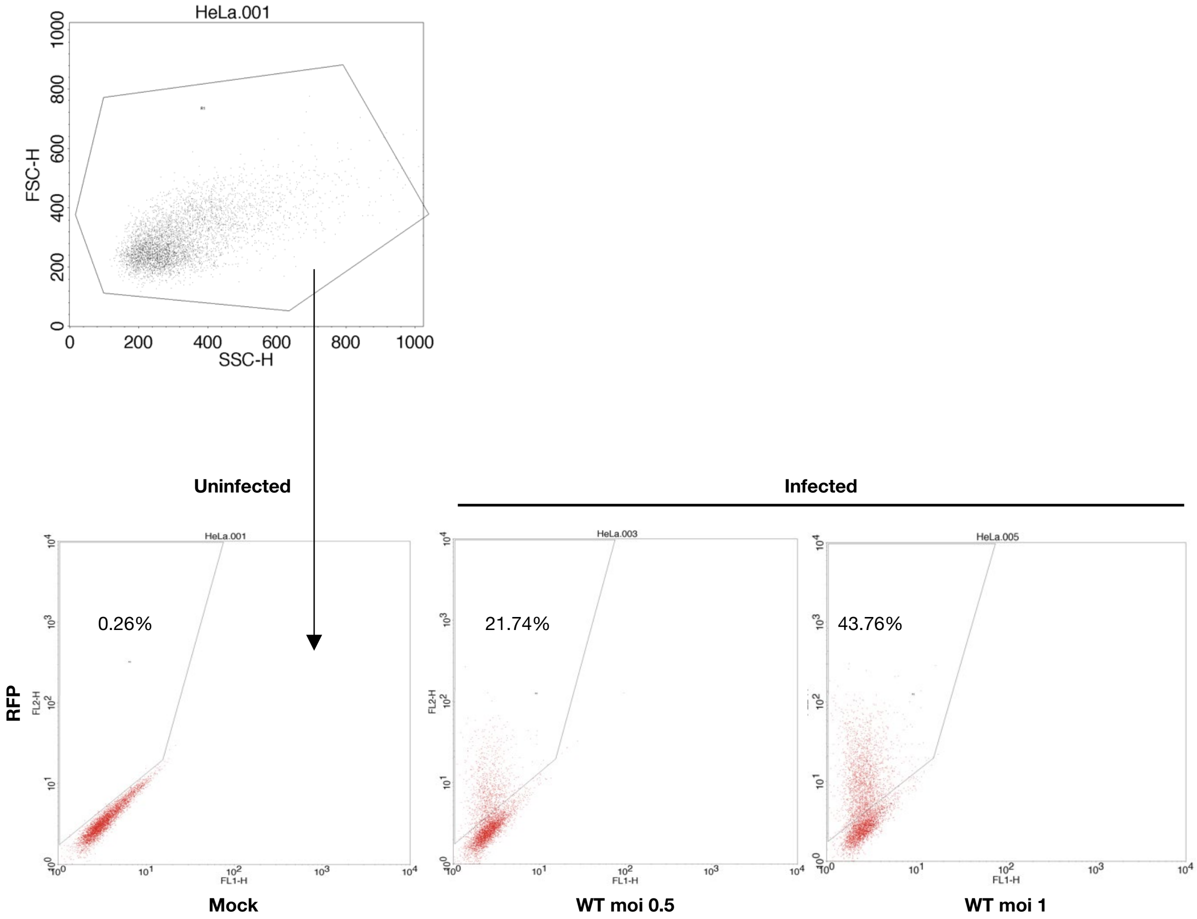

Supplementary Figure 10. Flow cytometry gating strategies. Live cells are gated (FSC vs. SSC plot). Representative dot plots demonstrating mock control and RFP positive cells. This gating strategy was applied to quantify all HIV-RFP infections presented in different figures.
