# Supplementary material for: The HIV-1 capsid core is an opportunistic nuclear import receptor
Source: Nat Commun. 2023 Jun 24;14:3782. doi: 10.1038/s41467-023-39146-5 (PMC10290713; doi:10.1038/s41467-023-39146-5)
Supplement: Supplementary file 2 — Reporting Summary [file 41467_2023_39146_MOESM2_ESM.pdf]

nature portfolio

Corresponding author(s): Vineet N. KewalRamani

Last updated by author(s): May 10, 2023

Reporting Summary

Nature Portfolio wishes to improve the reproducibility of the work that we publish. This form provides structure for consistency and transparency in reporting. For further information on Nature Portfolio policies, see our Editorial Policies and the Editorial Policy Checklist.

Statistics

For all statistical analyses, confirm that the following items are present in the figure legend, table legend, main text, or Methods section.

n/a	Confirmed	
☐	☒	The exact sample size (*n*) for each experimental group/condition, given as a discrete number and unit of measurement
☐	☒	A statement on whether measurements were taken from distinct samples or whether the same sample was measured repeatedly
☒	☐	The statistical test(s) used AND whether they are one- or two-sided<br>*Only common tests should be described solely by name; describe more complex techniques in the Methods section.*
☒	☐	A description of all covariates tested
☒	☐	A description of any assumptions or corrections, such as tests of normality and adjustment for multiple comparisons
☒	☐	A full description of the statistical parameters including central tendency (e.g. means) or other basic estimates (e.g. regression coefficient) AND variation (e.g. standard deviation) or associated estimates of uncertainty (e.g. confidence intervals)
☒	☐	For null hypothesis testing, the test statistic (e.g. *F*, *t*, *r*) with confidence intervals, effect sizes, degrees of freedom and *P* value noted<br>*Give P values as exact values whenever suitable.*
☒	☐	For Bayesian analysis, information on the choice of priors and Markov chain Monte Carlo settings
☒	☐	For hierarchical and complex designs, identification of the appropriate level for tests and full reporting of outcomes
☒	☐	Estimates of effect sizes (e.g. Cohen's *d*, Pearson's *r*), indicating how they were calculated

Our web collection on statistics for biologists contains articles on many of the points above.

Software and code

Policy information about availability of computer code

Data collection	BD FACSCalibur<br>Wallac 1450 MicroBeta TriLux Liquid Scintillation Counter & Lumi<br>DNA Engine Opticon (MJ Research, BioRad)<br>Deltavison deconvolve microscope (GE Healthcare)<br>DeltaVision OMX SR microscope (GE Healthcare)<br>Chemidoc XRS+ system (Bio-Rad)
Data analysis	BD CellQuest Pro (Version 6.0)<br>Softworks software (Version 7.0.0, GE Healthcare)<br>SoftWoRX 6.1 (Applied Precision)<br>GraphPad Prism 9 (Version 9.4.1)

For manuscripts utilizing custom algorithms or software that are central to the research but not yet described in published literature, software must be made available to editors and reviewers. We strongly encourage code deposition in a community repository (e.g. GitHub). See the Nature Portfolio guidelines for submitting code & software for further information.

Data

Policy information about availability of data

All manuscripts must include a data availability statement. This statement should provide the following information, where applicable:

- Accession codes, unique identifiers, or web links for publicly available datasets
- A description of any restrictions on data availability
- For clinical datasets or third party data, please ensure that the statement adheres to our policy

The authors declare that the data supporting the findings of this study are available within the paper and its supplementary information files. siRNA, shRNA, and gRNA sequences are provided in Materials. Source data are provided in a supplementary document.

Research involving human participants, their data, or biological material

Policy information about studies with human participants or human data. See also policy information about sex, gender (identity/presentation), and sexual orientation and race, ethnicity and racism.

Reporting on sex and gender	*Use the terms sex (biological attribute) and gender (shaped by social and cultural circumstances) carefully in order to avoid confusing both terms. Indicate if findings apply to only one sex or gender; describe whether sex and gender were considered in study design; whether sex and/or gender was determined based on self-reporting or assigned and methods used. Provide in the source data disaggregated sex and gender data, where this information has been collected, and if consent has been obtained for sharing of individual-level data; provide overall numbers in this Reporting Summary. Please state if this information has not been collected. Report sex- and gender-based analyses where performed, justify reasons for lack of sex- and gender-based analysis.*
Reporting on race, ethnicity, or other socially relevant groupings	*Please specify the socially constructed or socially relevant categorization variable(s) used in your manuscript and explain why they were used. Please note that such variables should not be used as proxies for other socially constructed/relevant variables (for example, race or ethnicity should not be used as a proxy for socioeconomic status). Provide clear definitions of the relevant terms used, how they were provided (by the participants/respondents, the researchers, or third parties), and the method(s) used to classify people into the different categories (e.g. self-report, census or administrative data, social media data, etc.) Please provide details about how you controlled for confounding variables in your analyses.*
Population characteristics	*Describe the covariate-relevant population characteristics of the human research participants (e.g. age, genotypic information, past and current diagnosis and treatment categories). If you filled out the behavioural & social sciences study design questions and have nothing to add here, write "See above."*
Recruitment	*Describe how participants were recruited. Outline any potential self-selection bias or other biases that may be present and how these are likely to impact results.*
Ethics oversight	*Identify the organization(s) that approved the study protocol.*

Note that full information on the approval of the study protocol must also be provided in the manuscript.

Field-specific reporting

Please select the one below that is the best fit for your research. If you are not sure, read the appropriate sections before making your selection.

☒ Life sciences ☐ Behavioural & social sciences ☐ Ecological, evolutionary & environmental sciences

For a reference copy of the document with all sections, see nature.com/documents/nr-reporting-summary-flat.pdf

Life sciences study design

All studies must disclose on these points even when the disclosure is negative.

Sample size	All experiments were performed in duplicate with reproducible data. Duplicates were performed in multiple different ways in different figure panels supporting reproducibility of results. Sample sizes were determined based on the authors' experience (Lee et al., Cell Host Microbe, 2010; Rebensburg et al., Nat Microbiol, 2021) and standard, generally accepted sample sizes in the field of virology (Kane et al., Nautre, 2013). siRNA screens were performed using two different commercially available siRNA pools (Dharmacon; Sigma) yielding similar results. Nup35 and POM121 are further characterized by shRNA and/or CRISPR knockout.
Data exclusions	Data on Nup35 and POM121 were not excluded.
Replication	All experiments were replicated at least two independent times. Some experiments were performed more than 5 times and verified using different methodologies, with results are consistent with the conclusions of the study.
Randomization	Immunofluorescence images were taken from random fields.

Blinding	None of the data were blinded as data were quantitative and measurements were made using relevant instruments and software.

Reporting for specific materials, systems and methods

We require information from authors about some types of materials, experimental systems and methods used in many studies. Here, indicate whether each material, system or method listed is relevant to your study. If you are not sure if a list item applies to your research, read the appropriate section before selecting a response.

Materials & experimental systems

n/a	Involved in the study
☐	☒ Antibodies
☐	☒ Eukaryotic cell lines
☒	☐ Palaeontology and archaeology
☒	☐ Animals and other organisms
☒	☐ Clinical data
☒	☐ Dual use research of concern
☒	☐ Plants

Methods

n/a	Involved in the study
☒	☐ ChIP-seq
☐	☒ Flow cytometry
☒	☐ MRI-based neuroimaging

Antibodies

Antibodies used

1 Antibodies used in Western blot:
Primary antibodies -
Mouse monoclonal anti-cyclophilin A antibody (Abcam, Cat. No. AB58144, clone 1F4-1B5) 1:3000 dilution
Rabbit polyclonal anti-CPSF6 antibody (Novusbio, Cat. No. NB100-61596) 1:3000 dilution
Rabbit polyclonal anti-Nup53 antibody (Abcam, Cat. No. ab126993) 1:1000 dilution
Rabbit polyclonal anti-Nup53 antibody (Bethyl, Cat. No. A301-781A) 1:2000 dilution
Rabbit polyclonal anti-Nup53 antibody (GeneTex, Cat. No. GTX64510) 1:1000 dilution
Rabbit polyclonal anti-Nup53 antibody (Novusbio, Cat. No. NB100-93322) 1:1000 dilution
Rabbit polyclonal anti-Nup93 antibody (Abcam, Cat. No. AB168805) 1:500 dilution
Mouse monoclonal anti-Nup153 antibody (Abcam, Cat. No. ab24700, clone QE5) 1:500 dilution
Rabbit polyclonal anti-Nup155 antibody (Abcam, Cat. No. ab73292) 1:500 dilution
Rabbit polyclonal anti-Nup188 antibody (Novusbio, Cat. No. NBP1-28748) 1:1000 dilution
Rabbit polyclonal anti-Nup205 antibody (Novusbio, Cat. No. NBP1-91247) 1:750 dilution
Rabbit polyclonal anti-Nup358 (RanBP2) antibody (Abcam, Cat. No. ab64276) 1:1000 dilution
Rabbit polyclonal anti-POM121 antibody (GeneTex, Cat. No. GTX102128) 1:500 dilution
Mouse monoclonal anti-TNPO3 antibody (MyBioSource, Cat. No. MBS120261) 1:200 dilution
Mouse monoclonal anti-HA antibody (Sigma, Cat. No. H9658-2ML, clone HA-7) 1:1500 dilution
Mouse monoclonal anti-tubulin antibody (Sigma, Cat. No. T6074, clone B-5-1-2) 1:50,000 dilution
Human Anti-Human Immunodeficiency Virus Type 1 Neutralizing Serum 1, Heat-inactivated (HIV reagent program, Cat. No. ARP-1984) 1:250 dilution

Secondary antibodies -
ECL Anti-mouse IgG, Horseradish Peroxidase linked whole antibody (from sheep) (GE Healthcare, Cat. No. NA931V) 1:10,000 dilution
ECL Anti-rabbit IgG, Horseradish Peroxidase linked whole antibody (from Donkey) (GE Healthcare, Cat. No. NA934V) 1:10,000 dilution

Antibodies used in immunofluorescence:
Primary antibodies -
Mouse monoclonal anti-cyclophilin A antibody (Abcam, Cat. No. AB58144, clone 1F4-1B5) 1:2000 dilution
Rabbit polyclonal anti-CPSF6 antibody (Novusbio, Cat. No. NB100-61596) 1:400 dilution
Mouse monoclonal anti-Nuclear Pore Complex antibody (BioLegend, Cat. No. 902907, clone MAb 414) 1:200 dilution
Rabbit polyclonal anti-Lamin B1 antibody (Abcam, Cat. No. ab16048) 1:2000 dilution

Secondary antibodies -
Goat anti-rabbit Alexa Fluor 488 antibody (Molecular Probes, Cat. No. A11008) 1:2000 dilution
Goat anti-mouse Alexa Fluor 488 antibody (Molecular Probes, Cat. No. A11017) 1:2000 dilution
Goat anti-mouse Alexa Fluor 546 antibody (Molecular Probes, Cat. No. A11019) 1:2000 dilution
Goat anti-rabbit Alexa Fluor 555 antibody (Molecular Probes, Cat. No. A21429) 1:2000 dilution

Validation

All antibodies except human anti-human immunodeficiency virus type 1 neutralizing serum1 (heat-inactivated) were validated by respective vendors.
Human Anti-Human Immunodeficiency Virus Type 1 Neutralizing Serum1 (heat-inactivated, ARP-1984) was obtained from NIH AIDS Reagent Program and validated. https://www.hivreagentprogram.org/Catalog/HRPPolyclonalAntiserum/ARP-1984.aspx
Mouse monoclonal anti-cyclophilin A antibody (Abcam, Cat. No. ab58144, clone 1F4-1B5) suitable for WB, ICC/IF, IP, and Flow Cyt. https://www.abcam.com/products/primary-antibodies/cyclophilin-a-antibody-1f4-1b5-ab58144.html
Rabbit polyclonal anti-CPSF6 antibody (Novusbio, Cat. No. NB100-61596) suitable for WB, Simple Western, ICC/IF, IHC, IHC-P, and IP. https://www.novusbio.com/products/cpsf6-antibody_nb100-61596
Rabbit polyclonal anti-Nup53 antibody (Abcam, Cat. No. ab126993) suitable for WB.

https://www.abcam.com/products/primary-antibodies/nup35-antibody-ab126993.html
Rabbit polyclonal anti-Nup53 antibody (Bethyl, Cat. No. A301-781A) suitable for WB, IHC, and IP. https://www.thermofisher.com/antibody/product/NUP35-Antibody-Polyclonal/A301-781A
Rabbit polyclonal anti-Nup53 antibody (GeneTex, Cat. No. GTX64510) suitable for WB. https://www.genetex.com/Product/Detail/Nup53-antibody/GTX64510
Rabbit polyclonal anti-Nup53 antibody (Novusbio, Cat. No. NB100-93322) suitable for WB, ICC/IF, IHC, IHC-P, and IP. https://www.novusbio.com/products/nup53-antibody_nb100-93322
Rabbit polyclonal anti-Nup93 antibody (Abcam, Cat. No. AB168805) suitable for WB and IP. https://www.labome.com/product/Abcam/ab168805.html
Mouse Monoclonal anti-Nup153 antibody (Abcam, Cat. No. ab24700, clone QE5) suitable for ICC/IF. https://www.abcam.com/products/primary-antibodies/nup153-antibody-qe5-ab24700.html
Rabbit polyclonal anti-Nup155 antibody (Abcam, Cat. No. ab73292) suitable for WB and IF. This item is discontinued.
Rabbit polyclonal anti-Nup188 antibody (Novusbio, Cat. No. NBP1-28748) suitable for WB. This item is discontinued.
Rabbit polyclonal anti-Nup205 antibody (Novusbio, Cat. No. NBP1-91247) suitable for WB, ICC/IF, IHC, IHC-P, and KD. https://www.novusbio.com/products/nup205-antibody_nbp1-91247
Rabbit polyclonal anti-Nup358 (RanBP2) antibody (Abcam, Cat. No. ab64276) suitable for WB, ICC/IF, and IP. https://www.abcam.com/products/primary-antibodies/ranbp2-antibody-ab64276.html
Rabbit polyclonal anti-POM121 antibody (GeneTex, Cat. No. GTX102128) suitable for WB, ICC/IF, and IHC-P. https://www.genetex.com/Product/Detail/POM121-antibody-N2N3/GTX102128
Mouse monoclonal anti-TNPO3 antibody (MyBioSource, Cat. No. MBS120261) suitable for ICC and WB. https://www.mybiosource.com/monoclonal-human-antibody/tnpo3/120261
Mouse monoclonal anti-HA antibody (Sigma, Cat. No. H9658-2ML, clone HA-7) suitable for ICC, IP, indirect ELISA, and WB. https://www.sigmaaldrich.com/US/en/product/sigma/h9658
Mouse monoclonal anti-Tubulin antibody (Sigma, Cat. No. T6074, clone B-5-1-2) suitable for WB. https://www.sigmaaldrich.com/US/en/product/sigma/t6074
ECL Anti-mouse IgG, Horseradish Peroxidase linked whole antibody (GE Healthcare, Cat. No. NA931V) suitable for WB. https://us.vwr.com/store/product/16776620/anti-igg-sheep-antibody-hrp-horseradish-peroxidase
ECL Anti-rabbit IgG, Horseradish Peroxidase linked whole antibody (GE Healthcare, Cat. No. NA934V) suitable for WB. https://us.vwr.com/store/product/16776610/anti-igg-donkey-polyclonal-antibody-hrp-horseradish-peroxidase.
Mouse monoclonal anti-cyclophilin A antibody (Abcam, Cat. No. AB58144, clone 1F4-1B5) suitable for WB, ICC/IF, IP, and Flow Cyt. https://www.abcam.com/products/primary-antibodies/cyclophilin-a-antibody-1f4-1b5-ab58144.html
Rabbit polyclonal anti-CPSF6 antibody (Novusbio, Cat. No. NB100-61596) suitable for WB, Simple Western, ICC/IF, IHC, IHC-P, and IP. https://www.novusbio.com/products/cpsf6-antibody_nb100-61596
Mouse monoclonal anti-Nuclear Pore Complex antibody (BioLegend, Cat. No. 902907, clone MAb 414) suitable for https://www.biolegend.com/en-us/products/purified-anti-nuclear-pore-complex-proteins-antibody-11498?GroupID=GROUP26
Rabbit polyclonal anti-Lamin B1 antibody (Abcam, Cat. No. ab16048) suitable for https://www.abcam.com/products/primary-antibodies/lamin-b1-antibody-nuclear-envelope-marker-ab16048.html
Goat anti-rabbit Alexa Fluor 488 antibody (Molecular Probes, Cat. No. A11008) suitable for IHC, ICC/IF, and Flow Cyt. https://www.thermofisher.com/antibody/product/Goat-anti-Rabbit-IgG-H-L-Cross-Adsorbed-Secondary-Antibody-Polyclonal/A-11008
Goat anti-mouse Alexa Fluor 488 antibody (Molecular Probes, Cat. No. A11017) suitable for ICC/IF and Flow Cyt. https://www.thermofisher.com/antibody/product/Goat-anti-Mouse-IgG-H-L-Cross-Adsorbed-Secondary-Antibody-Polyclonal/A-11017
Goat anti-mouse Alexa Fluor 546 antibody (Molecular Probes, Cat. No. A11019) suitable for ICC/IF. https://www.thermofisher.com/antibody/product/Goat-anti-Mouse-IgG-H-L-Cross-Adsorbed-Secondary-Antibody-Polyclonal/A-11019
Goat anti-rabbit Alexa Fluor 555 antibody (Molecular Probes, Cat. No. A21429) suitable for CC/IF and Flow Cyt. https://www.thermofisher.com/antibody/product/Goat-anti-Rabbit-IgG-H-L-Highly-Cross-Adsorbed-Secondary-Antibody-Polyclonal/A-21429

Eukaryotic cell lines

Policy information about cell lines and Sex and Gender in Research

Cell line source(s)	GHOST (Previously developed by the corresponding author and made available through the NIH sponsored HIV Reagent Program)<br>HEK293T (ATCC, CRL-3216)<br>HeLa (ATCC, CCL-2)<br>H9 (ATCC, HTB-176)<br>Jurkat (ATCC, TIB-152)<br>MT4 (gift of Eric Freed lab, NCI, NIH)
Authentication	Cell lines were not authenticated
Mycoplasma contamination	Cells tested negative for mycoplasma following the manufacturer's instructions for the MycoAlert Assay Control Set from Lonza.
Commonly misidentified lines<br>(See ICLAC register)	No commonly misidentified cell lines were used in the study. HeLa cells are often the contaminating cell for commonly misidentified cells.

Flow Cytometry

Plots

Confirm that:

☒ The axis labels state the marker and fluorochrome used (e.g. CD4-FITC).

☒ The axis scales are clearly visible. Include numbers along axes only for bottom left plot of group (a 'group' is an analysis of identical markers).

☒ All plots are contour plots with outliers or pseudocolor plots.

☒ A numerical value for number of cells or percentage (with statistics) is provided.

Methodology

Sample preparation	At several post-infection time points, cells were washed with PBS and treated with trypsin-EDTA. The detached cells were mixed 1:1 with 2% FCS in PBS and stored at 4°C until analysis on the flow cytometer.
Instrument	FACSCalibur (BD Biosciences)
Software	BD CellQuest Pro (version 6.0)
Cell population abundance	Sorted viable cell populations were >95%
Gating strategy	Live cells were gated based on FSC/SSC axes. A single color compensation control was collected for either GFP or RFP in the panel, then GFP+ or RFP+ cells were gated.

☒ Tick this box to confirm that a figure exemplifying the gating strategy is provided in the Supplementary Information.
